# Dual‐Biomimetic Bone Adhesive with Osteoimmunomodulatory Capabilities for Anatomical Reconstruction of Comminuted Fractures

**DOI:** 10.1002/advs.202501108

**Published:** 2025-07-08

**Authors:** Junyao Cheng, Hufei Wang, Ming Li, Jianpeng Gao, Xiao Liu, Chuyue Zhang, Pengfei Chi, Bo Li, Yuan Xve, Daoyang Fan, Zheng Wang, Jianheng Liu, Xing Wang, Licheng Zhang

**Affiliations:** ^1^ Department of Orthopedics Chinese PLA General Hospital Beijing 100853 China; ^2^ Chinese PLA Medical School Beijing 100853 China; ^3^ Beijing National Laboratory for Molecular Sciences Institute of Chemistry Chinese Academy of Sciences Beijing 100190 China; ^4^ University of Chinese Academy of Sciences Beijing 100049 China

**Keywords:** biocompatibility, bone adhesive, complex fracture, osteogenesis, robust adhesion

## Abstract

The development of bone adhesives capable of restoring natural bone structure and function represents a pioneering advancement in surgical technology, offering significant potential to improve the treatment outcomes of comminuted fractures. However, the development of a clinically demand‐driven adhesive, ensuring both reliable adhesion and osteogenic activity, poses a significant challenge. Herein, a structurally and functionally dual‐biomimetic bone adhesive is developed through the design of an organic–inorganic network‐enhanced hydrogel, which integrates caffeic acid‐grafted collagen (CAC), aminated laponite (ALAP), and N‐hydroxysuccinimide ester‐terminated polyethylene glycol (tetra‐PEG‐SC). Benefiting from nanosheet‐induced strengthening, the mechanically reinforced adhesive can maintain integrity under extreme compression (98%) and tensile (600%) deformations, facilitating anatomical repositioning of rabbit radius and porcine femur fractures. Of greater importance, the adhesive accelerated fracture healing, potentially via ROS scavenging and immunomodulation, as evidenced by reduced oxidative stress and M2 macrophage polarization in vitro. Assessed through an innovative rabbit radius comminuted fracture model, the injectable bone adhesive demonstrates rapid and flexible adhesion of bone fragments in a blood‐rich environment, as observed over the 2 fold improvement in biomechanical and radiological performance compared with commercially available cyanoacrylate adhesives. This intricately designed bone adhesive holds promise as a novel solution for addressing complex fracture cases in surgical treatment.

## Graphical abstract

A structurally and functionally dual bionic bone adhesive is engineered through an organic–inorganic cohesion strategy, which significantly reinforces the adhesive's bone tissue adhesion as well as osteogenic activity. In a pioneering rabbit radius comminuted fracture model, the bone adhesive exhibits exceptional capability in immobilizing bone fragments and expediting the healing process, demonstrating the great potential in addressing clinically complex fractures.

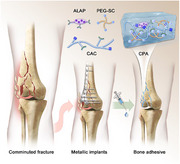

## 1 Introduction

Despite being one of the most common injuries to the musculoskeletal system, the optimal treatment of comminuted fractures is far from established and sparks extensive debate among trauma specialists.^[^ ^1^, ^2^, ^3^ ^]^ Internal fixation is recommended for the vast majority of comminuted fractures, with a range of techniques and implants available. However, there is concern regarding the complication rates, largely related to soft‐tissue injuries, fracture malreduction, instrumentation failure, and stress shielding, especially in those presenting with osteoporotic bone and/or periarticular fracture patterns.^[^ ^4^, ^5^, ^6^ ^]^ In the context of intra‐articular multifragmentary fractures, achieving a high‐standard anatomical reduction through internal fixation continues to pose a formidable challenge, resulting in a notable incidence of nonunion and atraumatic arthritis.^[^ ^7^, ^8^, ^9^ ^]^ Given these challenges and outcomes, there is a pressing necessity to explore and refine treatment strategies tailored to this high‐risk cohort.

The progress in material science and technology has opened up exciting possibilities for enhancing the surgical outcomes of complex fractures by employing bone adhesives to aid in fracture fixation and healing. Various materials, such as cyanoacrylate (CA), calcium phosphate cement, polyurethane, and bioactive glass, have been investigated for their potential in bone adhesion. However, these existing materials have fallen short of meeting the demands of clinical applications due to deficiencies in tissue adhesion, biocompatibility, biodegradability, or osteogenic activity.^[^ ^10^, ^11^, ^12^, ^13^, ^14^ ^]^ It follows that providing reliable adhesion while avoiding interference or even effectively promoting fracture healing is paramount for bone adhesives to be able to achieve translational clinical applications. Hydrogel‐based adhesives present distinctive advantages relative to conventional adhesives, primarily stemming from their remarkable diversity in material selection and design flexibility, thereby endowing them with significant potential to synergistically enhance adhesion properties, mechanical characteristics, and biofunctionality.^[^ ^15^, ^16^, ^17^ ^]^

The persistent challenge in bone adhesive development lies in the simultaneous optimization of material selection and design strategies to achieve effective fixation and accelerate healing of complex fractures. An exclusive focus on adhesive strength does not suffice for effective fracture repair; the presence of robust osteogenic activity is equally imperative. Primarily, biosafety of bone adhesives must be ensured, with vigilant consideration of potential adverse effects on biological metabolism and the in vivo microenvironment caused by their degradation products. Additionally, achieving the critical coordination between adhesive degradation and the healing process is imperative for promoting fracture repair. This necessitates that the adhesive degrades at an appropriate rate while maintaining stable osteogenesis activity. The construction of crosslinked networks using organic–inorganic materials offers a promising approach for the structural and functional optimization of bone adhesives.^[^ ^18^, ^19^, ^20^ ^]^ Laponite (LAP) has been demonstrated as an ideal inorganic component with physical network strengthening and valuable osteogenic properties.^[^ ^21^, ^22^, ^23^ ^]^ LAP's nanolayer structure boosts the cohesive energy within the hydrogel network, while its hydrophilicity and high specific surface area enable multifaceted interactions with biomolecules across various scales.^[^ ^24^, ^25^ ^]^ However, LAP is susceptible to lamellar aggregation owing to electrostatic interactions among particles.^[^ ^26^, ^27^ ^]^ This tendency impedes its uniform dispersion within the hydrogel network and limits its ability to fully leverage the network strengthening and bioactivity effects. On the other hand, the presence of a heightened inflammatory and oxidative stress microenvironment resulting from injury may cause organic–inorganic phase separation from rapid degradation of organic hydrogels, thus posing threats to both the structural integrity and osteogenic capability of bone adhesives.^[^ ^28^, ^29^ ^]^ Therefore, the organic–inorganic cross‐linking strategy promptly mitigates oxidative stress and endures the fracture microenvironment, facilitating the development of adhesives that coordinate the degradation‐healing process while consistently promoting osteogenesis.

**Figure 1:**
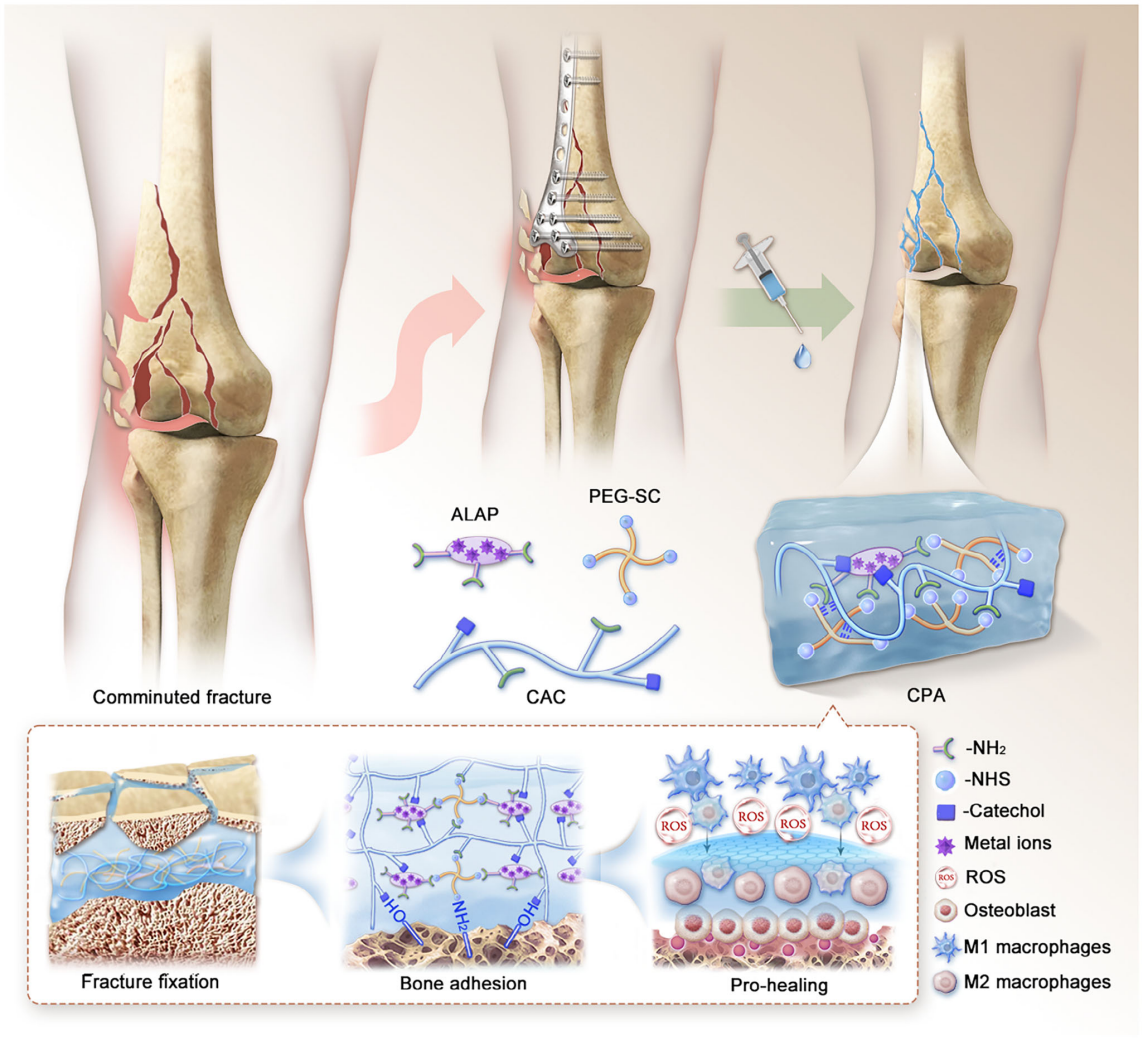
Schematic illustration for the preparation and characterization of the bone adhesive.

In this study, to develop an adhesive with the ability to securely stabilize fracture fragments and facilitate ongoing bone ingrowth, caffeic acid‐modified collagen (CAC) and N‐hydroxysuccinimide (NHS)‐encapsulated tetra‐armed PEG (tetra‐PEG‐SC) were employed as the foundational network components, and amine‐modified LAP (ALAP) was introduced to create a highly integrated organo–inorganic hydrogel with both structural and functional bionic characteristics. In addition to its role in improving underwater adhesion and suppressing the overexpression of reactive oxygen species (ROS), the introduction of caffeic acid is noteworthy for chelating with metal ions on the LAP via the catechol moiety. This promotes the uniform distribution of LAP and ensures the lasting effectiveness of its structural enhancement and biological functions (**Figure** **1**). The resulting bone adhesive, benefiting from nanoscale organic–inorganic structural reinforcement, demonstrated impressive mechanical strength and adhesive properties. It exhibited exceptional adhesion to in vitro fresh porcine bone, allowing for precise anatomical fracture repositioning, superior to traditional internal fixation methods. To validate its performance in vivo, we introduced a novel rabbit radius comminuted fracture model by the creation of three fracture segments through two lacerations. This challenging scenario required the adhesive to possess a comprehensive set of attributes, including biosafety, controlled degradability, reliable adhesion, and osteogenic qualities. The in vivo findings unequivocally demonstrated that the novel bone adhesive effectively stabilized the comminuted bone segments and consistently promoted the fracture repair through synergistic mechanisms of structural degradation‐healing and the intrinsic antioxidant system activation. Overall, this pioneering adhesive holds substantial promise for providing perspectives and strategies to address complex fractures in medical treatments.^[^ ^30^, ^31^ ^]^

## 2 Results and Discussion

### 2.1 Preparation and Characterization

#### 2.1.1 Chemical Characterization of CAC and ALAP

**Figure 2:**
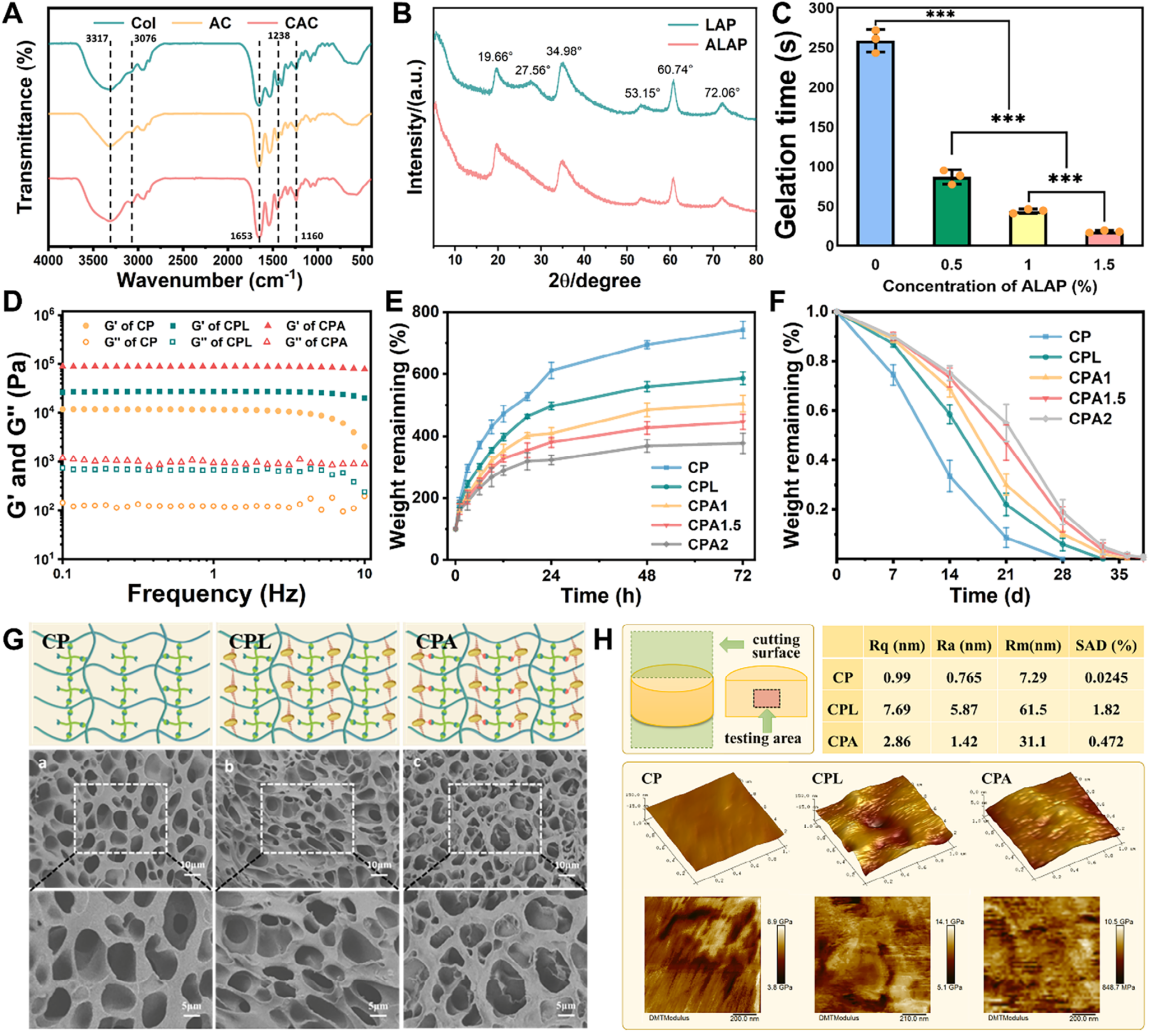
Characterization and structure of the bone adhesive. A) FT‐IR spectra of Col, AC, and CAC. B) XRD curves of LAP and ALAP. C) Gelation time of CPA hydrogels at different concentrations of ALAP (n = 3). D) Strain sweep of the hydrogels across the oscillation frequency range of 0.1–10 Hz. E) Swelling curves and F) degradation curves of the hydrogels (n = 3). G) Schematic structure and SEM images of the hydrogels, Scale bar: 10 µm (whole), 5 µm (inset). H) AFM topological scanning and Young's modulus tests of the hydrogels. (Data are presented as mean ± SD, ^***^ *p*< 0.001).

The synthesis of CAC involved a two‐step modification process. Initially, amination modification was employed to yield aminated collagen (AC), followed by grafting caffeic acid onto AC's amino group using carbodiimide chemistry. The primary amino group content was determined using the TNBS (2,4,6‐trinitrobenzenesulfonic acid) method. A standard calibration curve was established by reacting TNBS with β‐alanine solutions at varying concentrations (Figure S1, Supporting Information). Quantitative analysis revealed that the amino group content of native collagen (Col) was 0.12 mmol g^−1^, while that of the modified AC (aminocaproic acid‐conjugated collagen) significantly increased to 0.65 mmol g^−1^, validating the successful enhancement of amino functionality through chemical modification. According to the amino content after grafting modification in the AC and the integral area ratio of characteristic peaks (Ia/Ic‐e) in the 1H NMR spectra, the grafting ratio of caffeic acid was calculated as 4.5% (Figure S2, Supporting Information). FT‐IR was employed to examine the chemical structures of Col (collagen), AC, and CAC. The absorption peaks at 3317 and 3076 cm^−1^ corresponding to C─H stretching vibrations of the methylene, and 1238 and 1160 cm^−1^ belonging to the amide I and amide II bands, verified the successful condensation reaction between caffeic acid and the amine group. Furthermore, a characteristic peak at 1653 cm^−1^ was attributed to the aromatic C═C peak of the phenolic hydroxyl‐rich catechol moiety in CAC, reaffirming the chemical grafting of caffeic acid (**Figure** **2**A). TEM images revealed the “lamellar” morphology of LAP, with evident particle aggregation attributable to electrostatic forces (Figure S3a, Supporting Information). These LAP particles predominantly fell within the size range of 58.8–68.1 nm (Figure S3b, Supporting Information). The amino‐modification of LAP was clearly testified by the occurrence of typical C─H stretching vibration peaks at 3433 and 2934 cm^−1^ in FT‐IR (Figure S4, Supporting Information) and blurring or vanishing of originally sharp crystalline diffraction peaks in XRD (Figure 2B). While the XRD patterns of LAP and ALAP appeared similar, elemental analysis revealed a 9.6 fold increase in nitrogen content in ALAP (0.77% vs 0.08% in pristine LAP), confirming successful surface amination. The preserved crystalline structure indicates that the amino modification occurred predominantly on the nanoparticle surface without altering the core lattice, which is critical for maintaining LAP's inherent bioactivity while introducing functional groups for crosslinking. The hydrogen bonds between the amino groups and hydroxyl groups on the lamellar surface interfered with the original electrostatic force and induced the intermolecular irregular arrangement. The obtained ALAP with grafting amino groups could therefore establish the covalent cross‐links with NHS groups throughout the PEG network, providing a favorable mechanism for overcoming particle aggregation.

Upon mixing CAC and tetra‐PEG‐SC, both with or without LAP/ALAP, in a phosphate buffer solution (PBS), the resulting hydrogels of CAC/tetra‐PEG‐SC (CP), CAC/tetra‐PEG‐SC/LAP (CPL), and CAC/tetra‐PEG‐SC/ALAP (CPA) were rapidly formed at room temperature. Considering the gelation time and ease of operation, the tetra‐PEG‐SC content was fixed at 10% while the CAC concentration was varied between 5% and 12.5%. Consequently, the gelation time of CP ranged from 3.3 ± 0.2 to 9.2 ± 0.5 min (Figure S5, Supporting Information). The inclusion of ALAP significantly accelerated the gelation time for the CPA hydrogel, which could be attributed to the chemical coordination of Mg^2+^‐catechol and the presence of physical multi‐crosslinking sites provided by LAP nanoparticles. The gelation rate was crucial for operational feasibility, and with CPA, the gelation time can be customized to range from 0.5 to 3 min, ensuring ease of intraoperative manipulation (Figure 2C). The FT‐IR of CPA reveals several features: C─H stretching vibrations at 3412 and 2873 cm^−1^, N‐H stretching vibration at 1533 cm^−1^, as well as the appearance of absorption peaks at 1646 cm^−1^ and absence at 1742 cm^−1^ (Figure S6, Supporting Information). These spectral variations confirmed the formation of the CPA hydrogel network.

#### 2.1.2 Hydrogel Network Formation and Stability

The mechanical properties of bone adhesives were assessed using rheological testing. With a constant scanning frequency of 10 rad∙s^−1^, the stress exhibited a linear correlation with the strain within the range of 0.03–2%, and the modulus remained stable. When the strain was increased beyond 2%, the curves of the storage modulus (G*'*) and loss modulus (G″) intersected, indicating a transition of the adhesive from a viscoelastic solid phase to a liquid phase (Figure S7, Supporting Information). Across the oscillation frequency range of 0.1–10 Hz, G*'* consistently exceeded *G″*, indicating the adhesive's stable viscoelastic behavior. CPA demonstrated superior mechanical properties compared to CPL and CP, highlighting the effective enhancement of the hydrogel network strength due to the incorporation of ALAP (Figure 2F). Excessive swelling can disrupt the hydrogel structure and lead to a significant reduction in strength and adhesion properties. Therefore, controlling the swelling coefficient is crucial for ensuring the long‐term performance of bone adhesives.^[^ ^32^, ^33^ ^]^ As illustrated in Figure 2G, CPA exhibited a low swelling rate, primarily due to the reinforcement of the physical network by ALAP through multiple cross‐linking. This stable structure allowed CPA to degrade over a period of 5–6 weeks, aligning well with the fracture healing period (Figure 2H).^[^ ^34^, ^35^, ^36^ ^]^ To achieve the desired effects of cell proliferation and migration, the hydrogel network must exhibit an appropriate porous structure.^[^ ^37^ ^]^ SEM images provided insights into the microscopic morphology of CP, CPL, and CPA (Figure 2I). In comparison to CP with porous structures and smooth surfaces, CPL exhibited an uneven pore distribution and a rough surface. In contrast, CPA maintained a more uniform pore size with increased pore density. This indicated that the uniform distribution of ALAP in the gel network was notably superior to that of LAP, and ALAP, thus enhancing the cross‐linking density and reinforcing the network structure.

TEM images revealed distributed differences between LAP and ALAP nanoparticles. As shown in Figure S8 (Supporting Information), unmodified LAP exhibited pronounced particle aggregation due to interparticle electrostatic interactions. In contrast, amino‐functionalized ALAP demonstrated significantly improved dispersion within the hydrogel matrix, with reduced agglomeration and a more homogeneous spatial distribution. To further validate the uniformity of ALAP dispersion, energy‐dispersive spectroscopy (EDS) elemental mapping was employed to analyze the distribution of magnesium—a key component of LAP/ALAP. Figure S9 (Supporting Information) illustrates that the CPL hydrogel (containing 1.5% LAP) displayed localized magnesium clusters (yellow arrows), indicative of nanoparticle aggregation. Conversely, the CPA hydrogel (1.5% ALAP) exhibited a diffuse and uniform magnesium signal, confirming the enhanced dispersion achieved through amino modification and catechol‐metal coordination. AFM provides detailed surface structure information for further evaluating LAP/ALAP distribution within the hydrogel network. Due to the significant difference in the mechanical characteristics of the organic and inorganic phases, the distribution of Young's modulus measurement could help to determine the regional arrangement of LAP/ALAP (Figure 2J). The CP group, devoid of inorganic components, exhibited a flat morphology with a highly uniform modulus distribution, whereas CPL displayed notably increased surface roughness and concentrated high modulus regions. CPA, though less uniform than CP, outperformed CPL in modulus distribution and surface flatness. The significant roughness difference among the three hydrogels primarily arises from LAP/ALAP distribution disparities. ALAP, distributed via multiple cross‐linking, effectively counteracted the electrostatic forces between nanosheets, thereby facilitating the long‐term stabilization of the organic–inorganic network architectures. These findings collectively demonstrate that the amino‐functionalized modification of LAP, coupled with chelation‐driven integration into the hydrogel network, effectively mitigates nanoparticle aggregation while ensuring structural homogeneity.

### 2.2 Mechanical and Adhesive Properties

#### 2.2.1 Compression and Tensile Performance

**Figure 3:**
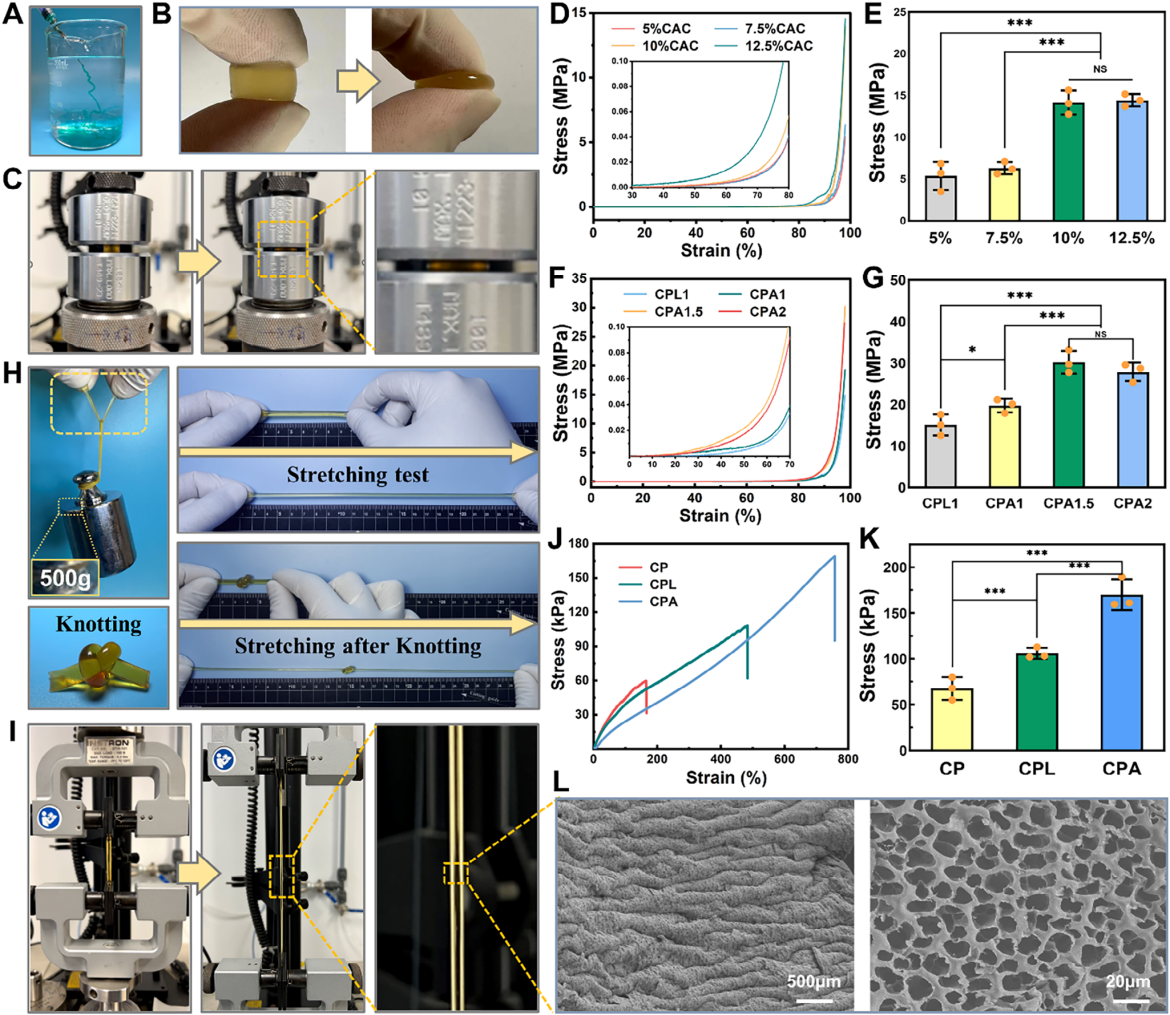
Structural and mechanical properties of the bone adhesive. A) Presentation of the injectability. B) Compressive observation of CPA hydrogel. C) Representative photographs of the hydrogels’ compression test. D) Compressive stress–strain curves and E) stress quantitative comparison of CP (n = 5). F) Compressive stress–strain curves and G) stress quantitative comparison of CPL and CPA (n = 5). H) Stretching test of the CPA following knotting and loading weights. I) Representative photographs of the tensile test. J) Tensile stress–strain curves and K) stress quantitative comparison of CP, CPL, and CPA (n = 5). L) SEM depiction of CPA in the stretched condition. (Data are presented as mean ± SD, ^*^ *p*< 0.05, ^**^ *p*< 0.01, ^***^ *p*< 0.001, NS: no significant difference).

Injectability and morphologic adaptability are key advantages of bone adhesives in fracture repair. Administered through minimally invasive channels, they minimize additional damage from overexposure.^[^ ^38^ ^]^ As demonstrated in **Figure** **3**A, the CPA hydrogel has good injectability and can be easily injected underwater. On account of the crucial roles of mechanical properties in dependable adhesion, particularly when applied to hard tissues, various forms of CPAs were prepared for mechanical assessment. As depicted in Figure 3B, the CPA adhesive withstood significant external extrusion forces without breaking. The compressive strength was assessed via a universal tensile machine. Remarkably, the bone binder's overall structure remained intact even under strains surpassing 90%, and its original shape could be fully restored after stress removal (Figure 3C). As the CAC concentration was gradually increased from 5% to 12.5%, the compressive strength of CP likewise increased (Figure 3D). When the concentration was 10% and 12.5%, the compressive strength reached 13.9 ± 1.5 MPa and 14.5 ± 1.2 MPa, respectively, with no statistically significant differences (Figure 3E). Considering the gelation time and preparation cost, 10% of CAC concentration was finally selected for follow‐up experiments. As anticipated, the inclusion of ALAP led to a significant enhancement in the compressive strength originating from the organic–inorganic network structure, which was synergistically reinforced by covalent bonding, hydrogen bonding, ionic bonding, and Mg^2+^‐catechol chelation. However, excessive inorganic components would potentially disrupt hydrogel networks and reduce mechanical properties by hindering cross‐linking.^[^ ^39^ ^]^ This explained the observed decline in compressive strength of CPAs at an ALAP concentration of 2% compared to 1.5% (Figure 3F,G).

Hydrogels comprising naturally derived materials often exhibit limited tensile strength, primarily due to the inherent weakness of these natural materials.^[^ ^40^ ^]^ However, it was a pleasant surprise to discover that CPA exhibited exceptional tensile strength in mechanical tests. When prepared in the form of strips (4 mm in diameter), CPA could withstand a weight load of 500 g (Figure 3H). CPA strips can be extended to more than 6 times their original length, and even when knotted, they can still be stretched several times. In the tensile mechanical tests (Figure 3I), the tensile strength of CPA reached an impressive 170 ± 3.3 kPa, which was significantly higher than that of CPL with 68 ± 8.9 kPa (Figure 3J,K). For SEM observation, the stretched CPAs were immersed and fixed in liquid nitrogen (Figure 3L). The stretched CPA displayed regularly arranged strips, and its porous structure maintained its integrity even under the highly stretched condition. The remarkable tensile properties of CPA are likely attributed to the effective dispersion of ALAP within the gel network. ALAP plays a pivotal role as a central element in network reinforcement, facilitating multiple cross‐linking and thereby strengthening the network. It also aids in overcoming electrostatic effects. Fatigue resistance is a critical factor for the long‐term and stable performance of bone adhesives in terms of their physical and biological efficacy.^[^ ^41^ ^]^ In cyclic compression and cyclic tensile tests, CPAs consistently maintained their morphological integrity (Figure S10, Supporting Information). In cyclic compression tests, CPA demonstrated excellent elastic recovery (with no permanent deformation) and exhibited a hysteresis energy loss of 10–15% (Figure S11, Supporting Information), indicating that it combines fatigue resistance with moderate energy dissipation capacity. This effectively mitigates the mechanical impact of physiological loads on the bone‐adhesive interface. Although CPA exhibits relatively low hysteresis energy loss (10–15%), its elastic recovery properties prevent interface loosening caused by plastic deformation, while moderate energy dissipation helps alleviate stress concentrations. Compared to fully rigid materials, this dual functionality better aligns with the application requirements of bone adhesives in dynamic physiological environments.

**Figure 4:**
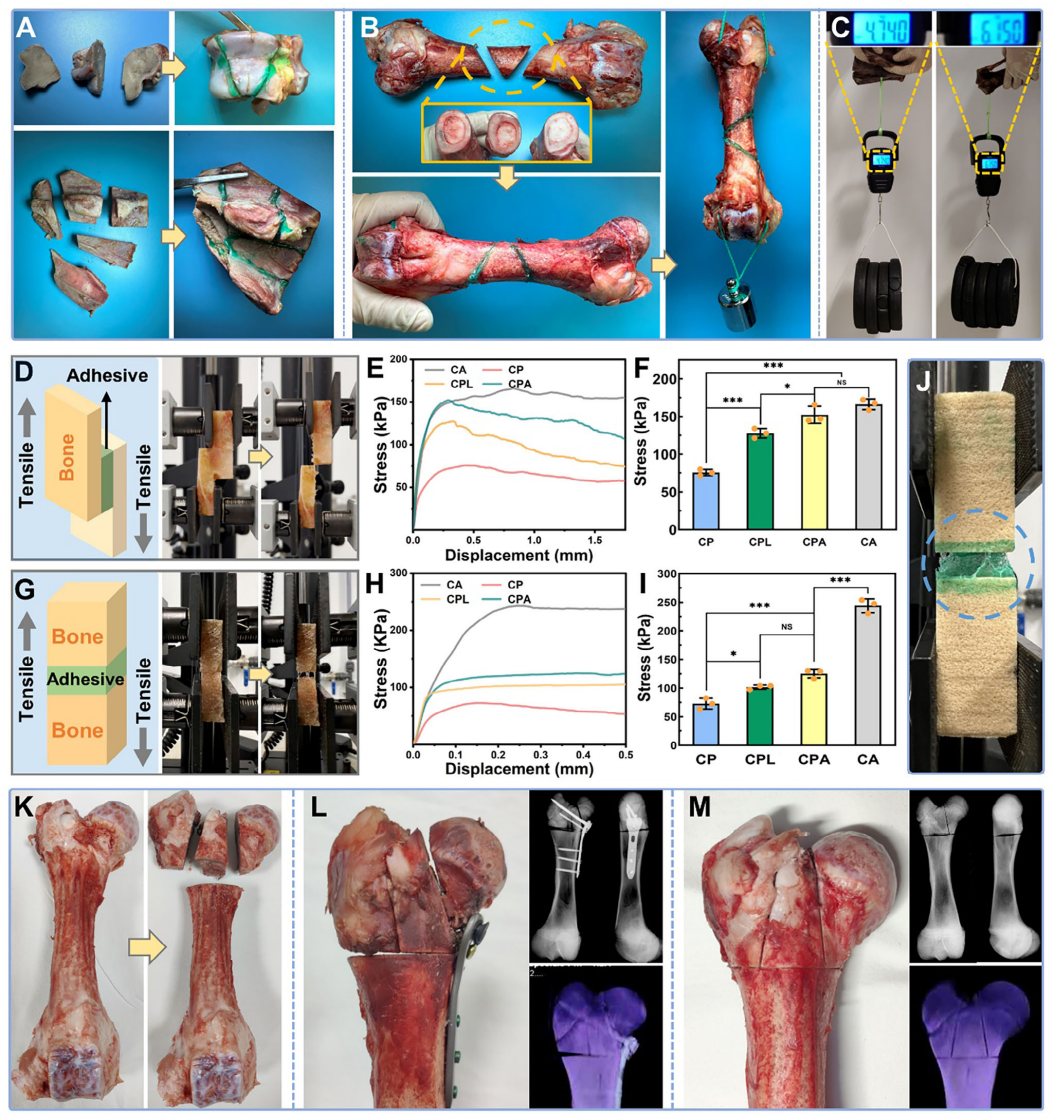
Characterization of the adhesive strength of the CPA hydrogels toward bones. A) Representative photographs of bone fragments adhesion. B) Representative photographs of femoral stem fracture adhesion. C) Weight‐bearing test after adhesion. D) Schematic and representative photographs of the lap‐shear mechanical test. E) Mechanical curve and F) stress quantification of lap‐shear mechanical test (n = 3). G) Schematic and representative photographs of the face‐to‐face mechanical test. H) Mechanical curve and I) stress quantification of face‐to‐face mechanical test. J) Morphological observation of CPA after adhesion failure. K) A model of comminuted fracture of the distal femur. L) Representative photographs of fracture repaired through internal fixation (n = 3). M) Representative photographs of a fracture repaired with bone adhesive. (Data are presented as mean ± SD, ^*^ *p*< 0.05, ^**^ *p*< 0.01, ^***^ *p*< 0.001, NS: no significant difference).

It is extremely difficult and time‐consuming to splice the bone fragments through traditional surgical methods, especially in cases of severe and highly comminuted fractures.^[^ ^42^ ^]^ In order to evaluate the potential of DPA hydrogel as a bone adhesive in different clinical situations, various in vitro experiments were performed. Fresh porcine bones were cut into multiple segments and repaired using CPA. As shown in **Figure** **4**A, the CPA was able to rapidly complete the integration of the broken bone fragments (the CPA was dyed green) with good alignment. More importantly, the self‐gravity force against the bone block could be overcome only 1 min after adhesion, and there was no fragmentation after being picked up with forceps. This is critical for saving valuable intraoperative time and reducing the risk of injury and infection.^[^ ^43^ ^]^ Multiple femoral stem fractures were consolidated using CPA, resulting in robust adhesion even in the presence of a reduced bonding surface and increased fragment weight (Figure 4B). The repaired porcine femur demonstrated effective resistance to gravitational forces in both shear and axial directions after 15 min of application. Furthermore, the weight‐bearing capacity of the isolated bonded bone remarkably reached 6.15 kg following a 12 h resting (Figure 4C). This underscores the efficacy of CPA in establishing robust and dependable bone adhesion within a reasonable timeframe, potentially enhancing the convenience and feasibility of fracture repair surgeries.

#### 2.2.2 Adhesive Strength in Simulated Clinical Scenarios

The adhesion properties of CP, CPL, and CPA were evaluated with a universal tensile machine, utilizing regular bone fragments obtained from fresh porcine bones. The bone fragments were initially adhered using a lap‐shear configuration (Figure 4D), with commercially available cyanoacrylate (CA) adhesive serving as the control material. The CA group exhibited the highest shear strength, reaching a peak stress of 166.1 ± 5.7 kPa. It is worth emphasizing that the CPA group demonstrated a shear strength closely comparable to that of the CA group, with a peak stress of 152.1 ± 7.3 kPa, and this difference was not statistically significant. Conversely, the shear strength of the CP and CPL groups was markedly lower than that of the former two, as illustrated in Figure 4E,F. Further testing was conducted using a face‐to‐face approach (Figure 4G). In this context, the tensile strength of the bone fragments bonded with CA reached 242.8 ± 14.1 kPa, markedly exceeding that of the hydrogel groups, as illustrated in Figure 4H,I. Notably, the CPA group exhibited a superior adhesion strength of 125.1 ± 6.2 kPa when compared to the CP and CPL groups. Examination of the bone fragments revealed a uniform adhesive film on the bone surface, and the adhesive's integrity remained unimpaired (Figure 3J; Figure S12, Supporting Information). It can be inferred that the fracture of the bonded bone fragments was caused by the adhesion failure under the extreme external forces, while the structural integrity of the bone adhesive remained intact at all times. Therefore, ALAP‐induced multiple cross‐linking substantially enhanced adhesion performance by strengthening the adhesive's inherent network, ensuring that the organic–inorganic polymer network constructed by ALAP maintained its structural stability even under the influence of powerful external forces. The ultimate tensile stress of CPA (170 ± 3.3 kPa) exceeds its face‐to‐face adhesive strength (125.1 ± 6.2 kPa), indicating that interfacial failure is attributed to the distinct mechanical loading modes. In bulk tensile tests, the hydrogel undergoes uniform stress distribution, while in adhesive shear tests, localized stress concentration at the hydrogel‐bone interface dominates. This discrepancy highlights that adhesive failure is governed by interfacial bonding dynamics rather than bulk material strength. The covalent crosslinking between NHS groups in tetra‐PEG‐SC and amino groups on bone surfaces enhances interfacial adhesion, yet localized shear forces under clinical scenarios may still exceed the interfacial bond strength before cohesive failure occurs.

Injuries to both bone and the surrounding soft tissues often involve substantial bleeding, and adhesive materials face challenges in functioning effectively within this liquid environment.^[^ ^44^ ^]^ The catechol moiety has been established as the primary functional component for underwater adhesion in mussels.^[^ ^45^, ^46^ ^]^ Similarly, within the CPA system, the incorporation of catechol‐enriched caffeic acid significantly enhances its adhesion on wet surfaces. When the bonded bone with CPA was exposed to a water stream, it was observed that the CPA adhered to the smooth cartilage surface could withstand the force of the water stream without detachment (Movie S1, Supporting Information). Subsequently, the CPA‐bonded bone was immersed in simulated body fluids and maintained strong adhesion even after 15 min (Movie S2, Supporting Information). The underwater adhesion performance was evaluated by simulating the in vivo body fluid circulation microenvironment through vertexing. Notably, the repaired femur exhibited consistent and stable adhesion throughout 15 min of continuous fluid agitation under weight‐bearing conditions (Movie S3, Supporting Information). The attainment of underwater adhesion by CPA can be primarily attributed to three key factors: 1) the mussel‐inspired adhesion properties of catechol moiety; 2) the rapid cross‐linking of NHS groups with amino groups on the side chains of bone surface proteins; 3) the prevention of structural damage caused by excessive expansion through ALAP‐facilitated reinforcement networks. To visually assess the effectiveness of bone adhesives in comparison to conventional internal fixation for fracture repair, a classical comminuted fracture of the distal femur was replicated with a porcine femur (Figure 4K). First, the fracture was fixed in vitro with standard fixation methods involving screws and plates, and the fracture reduction were assessed through X‐ray and CT tomography. As demonstrated in Figure 4L, despite the internal fixation procedure being conducted without time constraints and blood interference, noticeable gaps persisted between the fracture fragments after reduction. In contrast, fracture repair employing bone adhesive not only streamlined the process, saving time and effort, but also achieved the flawless alignment between the fracture fragments (Figure 4M). The small remaining gaps could only be detected by radiographic examination.

To address concerns regarding hydrogel swelling in physiological environments, we conducted post‐swelling mechanical tests under clinically relevant conditions. In practical fracture applications, hydrogel is exposed to limited blood/tissue fluid (Figure S13, Supporting Information), with most effusion occurring within 24 h post‐fracture.^[^ ^34^ ^]^ After 24 h of immersion in PBS (simulating the initial post‐fracture effusion phase), CPA retained >80% of its original compressive strength (31.0–25.5 MPa) and adhesive stress (155.2–127.0 kPa). This performance degradation aligns with practical scenarios, where CPA primarily encounters limited blood/tissue fluid exposure during the critical early adhesion phase. Furthermore, the optimized crosslinked network effectively counteracts swelling‐induced structural weakening, ensuring stable fixation even in dynamic physiological environments (Figure S14, Supporting Information). To validate the critical contribution of Mg^2^⁺‐catechol coordination to network reinforcement, CPA hydrogels were synthesized with Mg^2^⁺‐depleted ALAP treated with Ethylene diamine tetra acetic acid (EDTA) (CPA‐EDTA), and their mechanical properties and bone adhesion capabilities were rigorously compared with those of standard CPA. As shown in Figure S15 (Supporting Information), the removal of Mg^2^⁺ through EDTA treatment resulted in a 48.7% reduction in compressive strength (27.9–14.3 MPa) and a 61.2% decrease in adhesive stress (155.2–60.2 kPa). These results unequivocally demonstrate that Mg^2^⁺‐mediated chelation synergistically enhances the mechanical integrity of the hydrogel by stabilizing the dynamic crosslinking network through strong cation‐catechol interactions. This mechanism not only reinforces the structural resilience under cyclic loading but also optimizes interfacial adhesion to bone surfaces, highlighting its essential role in achieving clinically relevant performance for comminuted fracture repair.

### 2.3 In Vitro Biological Functions

#### 2.3.1 Cytocompatibility and Hemocompatibility

**Figure 5:**
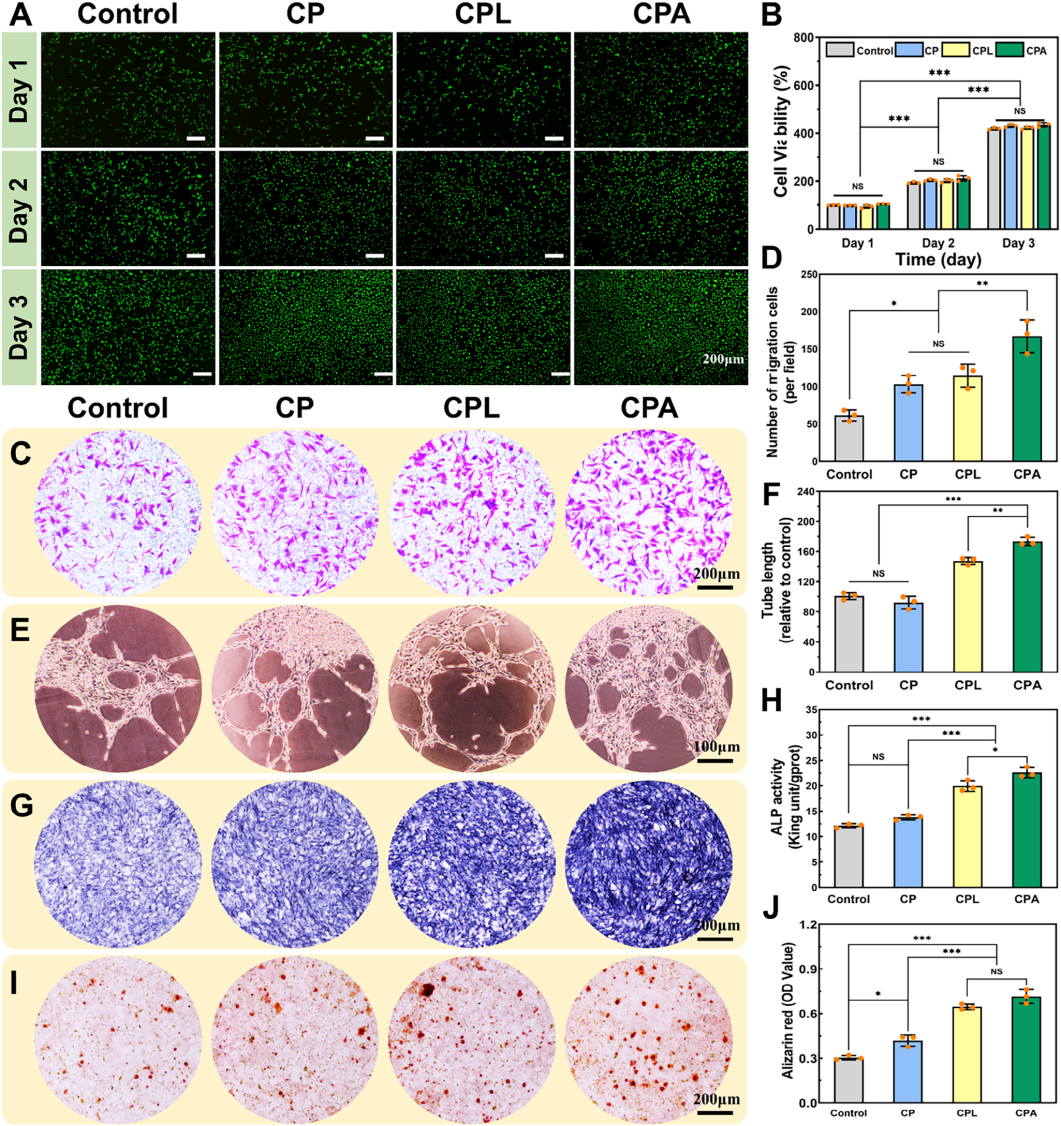
Evaluation of the in vitro biocompatibility and the osteogenic ability of the CPA hydrogels. A) Live/Dead staining and CCK‐8 (NIH 3T3 fibroblasts), Scale bar: 200 µm (n = 3). B) Cytotoxicity analysis (n = 3). C) Representative photographs and D) quantitative graph of the transwell experiment (HUVECs, n = 3). E) Representative photographs and F) quantitative graph of the angiogenesis experiment (HUVECs, n = 3). G) Representative photographs (HUVECs, n = 3) and H) quantitative graph of Alkaline phosphatase (ALP) staining (BMSCs, n = 3). I) Representative photographs and J) quantitative graph of alizarin red staining (BMSCs, n = 3). (Data are presented as mean ± SD, ^*^ *p*< 0.05, ^**^ *p*< 0.01, ^***^ *p*< 0.001, NS: no significant difference).

Biocompatibility is an essential criterion for bone adhesives, as it should facilitate cell proliferation and support harm‐free fracture healing.^[^ ^47^ ^]^ Initially, the cytocompatibility of the bone adhesives was evaluated using live/dead staining. As depicted in **Figure** **5**A, all hydrogel groups displayed a cell growth pattern similar to that of the control group, with nearly all NIH 3T3 fibroblasts exhibiting a healthy, spindle‐shaped morphology stained in green (indicating live cells). Quantitative assessment of cell viability through a CCK‐8 assay demonstrated a significant increase in cell proliferation within three days in the hydrogel group. By the third day of incubation, cell viability across all groups exceeded 400%, with no significant difference compared to the control group (Figure 5B). This favorable cytocompatibility can be attributed to the inherent cytophilicity of collagen in the hydrogel and the excellent cell adhesion and antioxidant properties of the catechol moiety.^[^ ^48^, ^49^ ^]^ Hemocompatibility is another vital safety consideration in biomaterial development. Erythrocyte suspensions from SD rats were obtained and co‐incubated with the hydrogels (Figure S16a, Supporting Information), depicting the appearance of the centrifuged supernatants. All hydrogel groups exhibited clarity and a light‐yellow color due to the presence of collagen, which markedly differentiated them from the positive control group (Triton X‐100). Quantitative analysis revealed that the hemolysis rate remained below 2% in all hydrogel groups, demonstrating excellent hemocompatibility of the bone adhesive (Figure S16b, Supporting Information).

#### 2.3.2 Antioxidant and Osteogenic Activity

The excessive production of free radicals during bone healing can impede the repair process, and elevated levels of ROS are one of the primary contributors to uncontrolled inflammation.^[^ ^13a^ ^]^ Consequently, ensuring antioxidant activity has become a crucial consideration in the design of bone adhesives. Caffeic acid, derived from plants, boasts potent antioxidant properties attributed to its abundance of phenolic hydroxyl groups. Therefore, grafting caffeic acid onto bone adhesives has the potential to imbue them with free radical scavenging capabilities.^[^ ^50^ ^]^ To evaluate the in vitro antioxidant activity of the bone adhesives, a DPPH scavenging assay was employed (Figure S17, Supporting Information). The hydrogel groups exhibited a DPPH scavenging capacity exceeding 90%, a level closely akin to that of the standard antioxidant, vitamin C (97%). Importantly, there were no significant differences observed among the groups, indicating that the DPPH scavenging ability of CPL and CPA remained unaffected, despite the catechol moiety's chelation with Mg^2+^ in LAP/ALAP.

From a bone tissue engineering perspective, an ideal bone adhesive should act as a dynamic osteogenic scaffold, creating a specialized environment that fosters and accelerates fracture healing. The reestablishment of blood microcirculation is a pivotal factor in facilitating metabolic processes at the fracture site.^[^ ^51^ ^]^ As such, the angiogenic activity of bone adhesives was assessed to evaluate their osteogenic efficacy. In the cell migration assay, the hydrogel groups demonstrated significantly higher migration rates compared to the control group, with the highest migration rate observed in the CPA group (Figure 5C,D). This enhanced migration can be attributed to the cell‐recruiting effects of magnesium, silicon, and catechol.^[^ ^52^, ^53^ ^]^ In the angiogenesis experiment using human umbilical vein endothelial cells (HUVECs), a mere few tubular structures were observed in the control group after 6 h of incubation (Figure 5E). In contrast, a higher number of tubular structures were formed in both the CPL and CPA groups. The early osteogenic induction activity of bone adhesives was assessed through ALP staining using bone marrow mesenchymal stem cells (BMSCs). As depicted in Figure 5G, in comparison to the control group, both the hydrogel group and CPA group displayed a more pronounced staining. Consistent with the ALP staining outcomes, alizarin red staining of BMSCs also underscored the substantial osteoinductive activity advantage of CPA. It can be inferred that the uniformly distributed ALAP displayed superior biological effects, leading to a substantial enhancement in the angiogenic activity of the CPA adhesive.

ALP activity is closely associated with the formation of calcium nodules in osteogenic processes and serves as a pivotal indicator for monitoring bone formation and metabolism maintenance.^[^ ^54^ ^]^ The early osteogenic induction activity of bone adhesives was assessed through ALP staining. As depicted in Figure 5G, in comparison to the control group, both the hydrogel group and CPA group displayed a more pronounced staining. This suggested the presence of localized active calcium deposition, potentially attributed to the calcium ion adsorption effect of the catechol moiety. Notably, the CPA group exhibited the most prominent positive staining areas, indicating an enhancement in osteogenic activity, likely associated with the uniform distribution of ALAP (Figure 5H). Consistent with the ALP staining outcomes, alizarin red staining also underscored the substantial osteoinductive activity advantage of CPA. The CPA group exhibited a notable abundance of red calcified nodules distributed in clusters, surpassing the other three groups in terms of both quantity and volume (Figure 5I,J). Two aspects need to be emphasized when talking about the osteogenic activity of CPA. First, the catechol moiety effectively recruits calcium ions, serving as nucleation sites for hydroxyapatite formation. Second, ALAP plays a crucial role in seamlessly integrating the organic–inorganic network structure, promoting osteoblast proliferation and differentiation, and expediting bone matrix formation by enhancing the release of magnesium ions.

### 2.4 In Vitro Study of Osteogenesis Mechanisms

#### 2.4.1 Bioinformatics Analysis of Osteogenic Pathways

**Figure 6:**
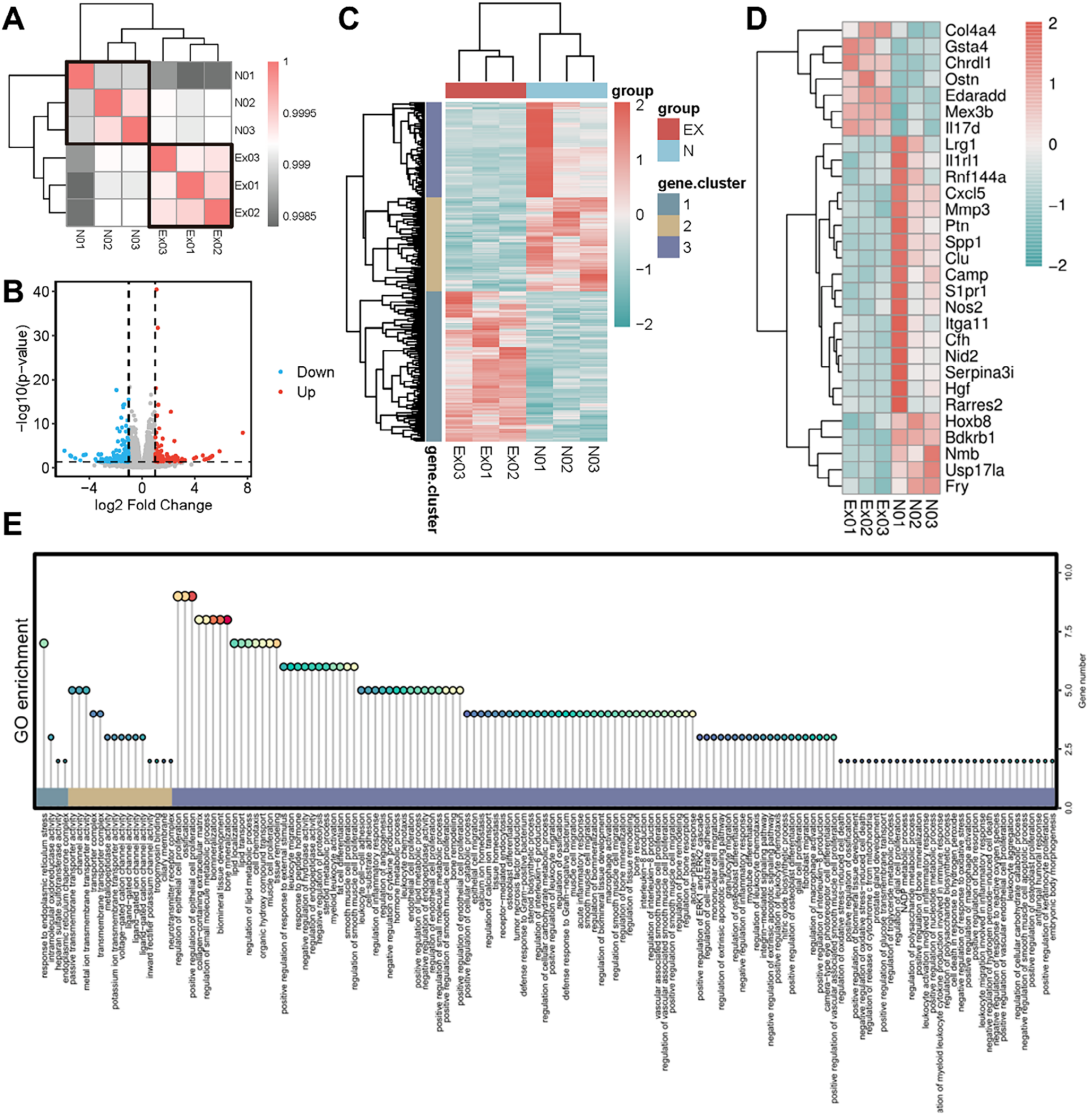
Exploration of gene expression patterns and functional enrichment analysis in BMSCs. A) Pearson's correlation analysis. B) Volcano plot analysis. (|log2FC| > 1, ^*^ *p*< 0.05) C) Cluster analysis of differentially expressed genes. D) Heatmap of immune‐related differentially expressed genes. E) GO enrichment pathway analysis. (n = 3).

To delve deeper into the mechanisms by which CPA facilitates bone regeneration and supports processes associated with bone healing, a bioinformatics analysis was performed on BMSCs. Pearson correlation analysis revealed distinct gene expression profiles between CPA‐treated BMSCs and controls (**Figure** **6**A). Volcano plots identified 299 differentially expressed genes (DEGs) (**|**log2FC**|** > 1, *p*< 0.05), with 132 upregulated and 167 downregulated (Figure 6B). Cluster analysis further highlighted the divergence in transcriptional regulation, particularly in pathways related to oxidative stress response and immune modulation (Figure 6C). Notably, we identified numerous genes linked to inflammatory factor regulation, inflammatory expression, and oxidative stress response among these differentially expressed genes, implicating immune‐environmental regulation in CPA‐induced osteogenesis (Figure 6D). GO enrichment analysis further underscored the activation of pathways critical for bone remodeling, including “osteoblast differentiation”, “extracellular matrix organization”, and “response to oxidative stress” (Figure 6E). These findings align with recent studies demonstrating that biomaterials modulating ROS levels can enhance BMSC osteogenesis by stabilizing HIF‐1α and activating Wnt/β‐catenin signaling.^[^ ^55^ ^]^ Additionally, the enrichment of “leukocyte migration” pathways correlates with CPA's ability to recruit immune cells, as evidenced by transwell assays. This immunomodulatory effect mirrors reports where magnesium ions released from biomaterials polarized macrophages toward an M2 phenotype, synergistically enhancing BMSC differentiation and angiogenesis.^[^ ^56^ ^]^

By integrating transcriptional profiling with functional assays, our analysis establishes a comprehensive framework for CPA's osteogenic efficacy: the adhesive not only provides structural support but also actively reprograms BMSCs to overcome oxidative and inflammatory barriers, thereby accelerating fracture healing. This dual functionality positions CPA as a paradigm‐shifting solution for complex fractures, where traditional adhesives fail to address the dynamic interplay between mechanical stability and biological repair. These findings align with recent studies emphasizing the critical role of immune modulation in bone regeneration. For instance, Zhang et al. demonstrated that biomaterials capable of suppressing M1 macrophage polarization while promoting M2 transition significantly accelerated fracture healing by reducing pro‐inflammatory cytokines (e.g., TNF‐α, IL‐6) and enhancing osteoblast differentiation.^[^ ^57^ ^]^ Similarly, our observation of enriched leukocyte migration pathways correlates with the work of Chen et al., who highlighted the importance of immune cell recruitment in establishing a pro‐regenerative microenvironment.^[^ ^58^ ^]^ The dual antioxidant and immunomodulatory effects of CPA, driven by caffeic acid and ALAP, mirror the synergistic strategies reported in advanced biomaterials. For example, Liu et al. developed a catechol‐functionalized hydrogel that scavenged ROS and promoted M2 polarization, leading to enhanced angiogenesis and osteogenesis.^[^ ^59^ ^]^ These parallels underscore CPA's potential to address the dual challenges of oxidative stress and chronic inflammation—key barriers in complex fracture repair.

#### 2.4.2 Macrophage Polarization and Immune Regulation

To further elucidate the potential osteoinductive mechanisms uncovered through bioinformatic analysis, an in‐depth in vitro study was conducted with mouse bone marrow‐derived macrophages (BMDM). Macrophages play a critical role in bone healing by modulating inflammation. Pro‐inflammatory M1 macrophages (marked by CD68) exacerbate oxidative stress and impede osteogenesis, while anti‐inflammatory M2 macrophages (marked by CD206) promote tissue repair and osteogenic differentiation. The mRNA expression was evaluated following the co‐cultivation of BMDM with hydrogels. The CPA groups displayed varying degrees of downregulation in CD68, a representative marker of M1 macrophages, along with an increase in the expression of CD31 and CD206, representative markers of M2 macrophages (Figure S18, Supporting Information). In contrast, the CP and CPL groups did not exhibit substantial anti‐inflammatory polarization. The CP group showed only a slight elevation in M2 markers, while the CPL group showed improvement but still maintained significantly lower levels compared to the CPA group. Immunofluorescence staining revealed CD68 overexpression in the control group, while the expression of CD31 and CD206 was notably diminished (Figure S19, Supporting Information). Importantly, the addition of caffeic acid alongside ALAP facilitated a substantial reduction in M1 marker expression while concurrently enhancing the expression of M2 markers. The findings above indicated that CPA effectively impeded initial M1 polarization during the acute inflammatory phase, while simultaneously reshaping the immune microenvironment by promoting M2 polarization. This anti‐inflammatory effect primarily arises from the biological properties of catechol and ALAP. The synergistic interaction of both components amplified the immunomodulatory effects, thereby enabling CPA to effectively promote osteogenesis by mitigating excessive inflammatory responses. This immunomodulatory effect synergizes with the adhesive's intrinsic antioxidant properties (Figure S16, Supporting Information) to accelerate fracture healing.

The observed M2 polarization and IL‐10 upregulation in CPA‐treated macrophages are consistent with emerging paradigms in immunomodulatory biomaterials. Recent studies on magnesium ions have demonstrated their concentration‐dependent effects on osteoblast behavior through activation of the PI3K/Akt signaling pathway.^[^ ^60^ ^]^ Our results extend this understanding by demonstrating that ALAP's amino‐modified surface enhances ion release kinetics, ensuring sustained Mg^2^⁺ bioavailability. Furthermore, the catechol moiety's role in ROS scavenging complements ALAP's immunomodulatory effects, creating a balanced microenvironment conducive to bone repair. This dual‐action mechanism contrasts with traditional single‐functional materials, such as polyurethane‐based adhesives, which often lack bioactivity.^[^ ^61^ ^]^ However, challenges remain in achieving long‐term immune homeostasis. For instance, Spiller et al. noted that excessive M2 polarization might lead to fibrotic encapsulation, highlighting the need for precise control over macrophage dynamics.^[^ ^62^ ^]^ Future studies could optimize CPA's degradation profile to align with the temporal requirements of inflammation resolution and tissue remodeling.

### 2.5 In Vivo Fracture Repair

**Figure 7:**
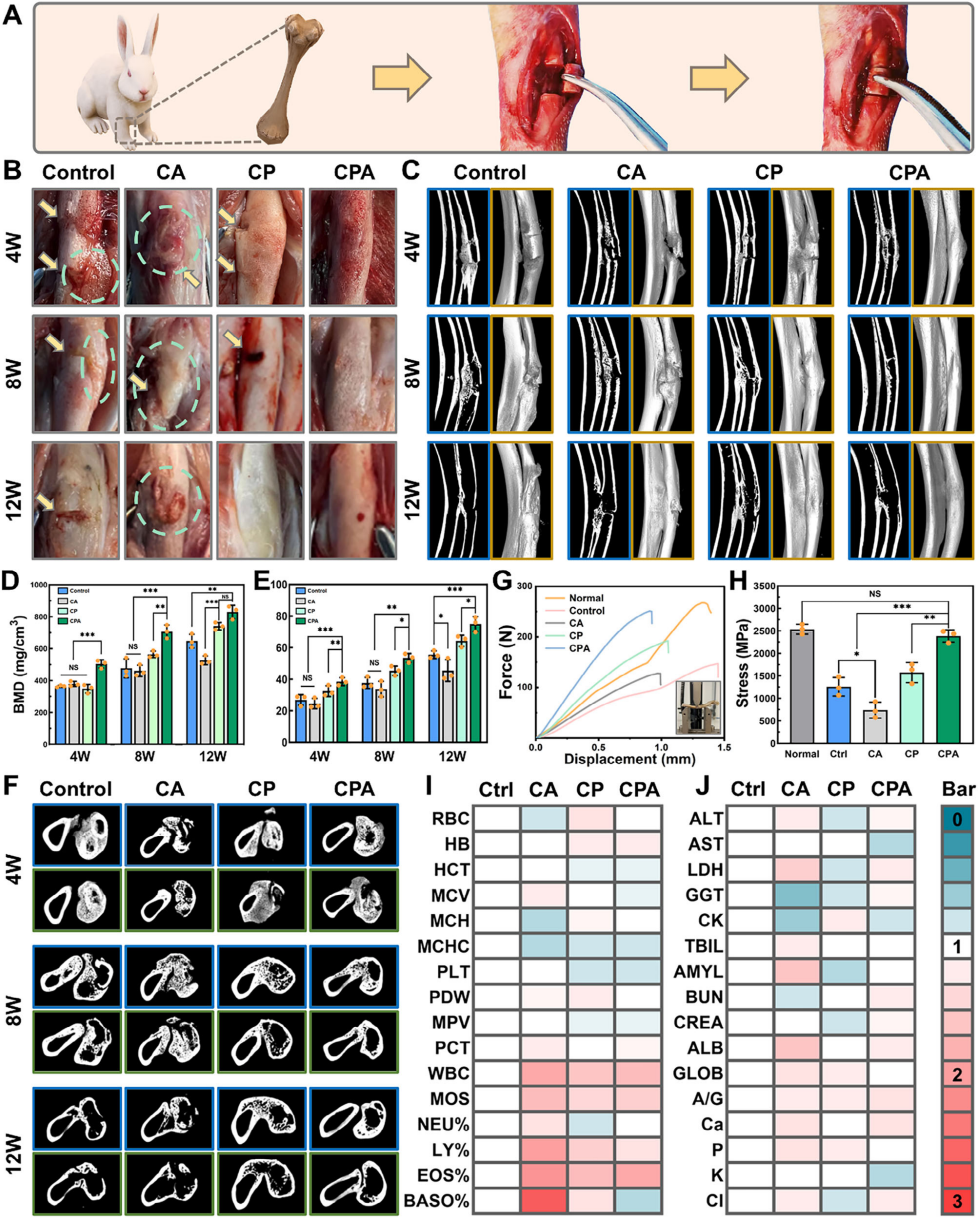
In vivo evaluation of the CPA hydrogels for repairing comminuted radial fractures in rabbits. A) Schematic illustration of the rabbit radius fracture model. B) Representative photographs depicting the appearance of fracture repair (yellow arrows indicating fracture breaks; green circles marking abnormal bone growth). C) Coronal tomographic images (denoted by the blue border) and 3D reconstructed images (denoted by the yellow border) acquired from CT scans at 4, 8, and 12 weeks. D) Quantitative assessment of bone mineral density (BMD) in the fracture region at 4, 8, and 12 weeks (n = 3). Quantitative assessment of BV/TV in the region of interest (ROI) at 4, 8, and 12 weeks (BV: the volume of new bone, TV: the volume of ROI, n = 3). F) CT transverse images at 4, 8, and 12 weeks postoperatively (Blue and green borders denoting proximal and distal fracture sections, respectively). G) Photographs of radius specimens undergoing biomechanical testing and corresponding force‐displacement curves at the 12th week. (The “Normal group” represents undamaged radius specimens from rabbits of the same age for comparison). H) Quantitative assessment for biomechanical testing (n = 3). I) Whole‐blood analysis and J) comprehensive metabolic screening of the rabbit after implantation with CA, CP, and CPA. Values were normalized to those for the control group (Blood test indicators are detailed in the supporting information, n = 3). (Data are presented as mean ± SD, ^*^ *p*< 0.05, ^**^ *p*< 0.01, ^***^ *p*< 0.001, NS: no significant difference).

Previous studies on bone repair have employed diverse animal models to evaluate biomaterial efficacy. For instance, calvarial defect models in rodents are widely used for cranial bone regeneration due to their simplicity and standardized defect size, yet they fail to replicate the biomechanical complexity of load‐bearing fractures. Similarly, femoral condyle defects in rabbits or rats, while providing insights into osteochondral repair, often involve non‐comminuted fractures that lack the fragmentation and instability characteristic of clinical comminuted fractures. Notably, existing rabbit radius fracture models, such as transverse or segmental defects, typically generate only two fragments, oversimplifying the multifragmentary nature of severe comminuted fractures. In contrast, our study introduces a novel rabbit radius comminuted fracture model with three unstable bone fragments, mimicking the clinical complexity of comminuted fractures. **Figure** **7**A provided a schematic representation of the fracture model, where, following two incisions, the rabbit radius generated three unstable comminuted bone fragments.

The control group included CA, renowned for its superior adhesive properties, as well as a blank control where the resected bone fragments were reset directly without any adhesive application. Morphological observations of the fracture area were conducted at 4, 8, and 12 weeks postoperatively (Figure 7B). Both the CP and CPA groups exhibited a trend toward fracture healing and remodeling at 4 weeks postoperatively. Notably, in comparison to the CP group, the CPA group demonstrated accelerated and superior fracture repair, achieving complete bone healing by the 8th week. This aligns with recent studies emphasizing the importance of bioactive and degradable adhesives in fracture repair. For instance, Cui et al. reported that GelDex‐AMBGN adhesive achieved synchronized degradation and osteogenesis in rabbit radius fractures, with new bone volume (BV/TV) doubling compared to non‐degradable controls.^[^ ^63^ ^]^ Similarly, Xu et al. demonstrated that bioactive glass‐incorporated adhesives facilitated 2 fold higher calcium deposition in mouse cranial defects compared to cyanoacrylate, underscoring the critical role of bioactivity in accelerating healing.^[^ ^64^ ^]^

In contrast, the control group experienced significant bone fragment displacement due to the absence of effective fixation. Furthermore, extensive peripheral osteophytes were observed in the control group, potentially arising from cycles of new bone reconstruction and destruction. Remarkably, identifiable fracture sections (indicated by yellow arrows) were still evident in the control group at 12th week, signifying a substantial delay in the fracture healing process. This underscored the pivotal role of dependable fixation in the context of fracture healing. In the CA group, the bone tissue exhibited extensive adhesion to the surrounding tissues. The irregular surface of the bone tissue and the presence of substantial reactive hyperplasia (highlighted by green circles) had a detrimental impact on the bone healing process. The persistent non‐degradability of CA adhesives mirrors findings from Yang et al.,^[^ ^65^ ^]^ where residual CA formed mechanical barriers that disrupted osteoblast migration and trabecular bridging in rat femoral defects.

Radiographic examination of the fracture site was conducted (Figure S20, Supporting Information). Notably, there was substantial displacement of bone fragments (highlighted by yellow circles) in the control group at the 4th week. Due to the inadequate fixation, the newborn bone trabeculae were not effectively protected, leading to a detrimental cycle of generation and disruption. The delayed healing resulted in the continued visibility of the fracture line in the control group at the 12th week postoperatively. In the CA group, observations unveiled evident indications of bone nonunion, with the defect area (highlighted by green circles) remaining at 12 weeks. This outcome can be attributed to CA's limited degradability. The persistence of CA at the fracture site formed a physical barrier that obstructed new bone growth, ultimately resulting in bone nonunion. This aligns with reports by Tang et al.,^[^ ^63^ ^]^ where non‐degradable polyurethane adhesives caused delayed healing in rabbit radial fractures. Conversely, the CPA group exhibited a remarkable enhancement in both the degree and quality of healing. The bone fragments were well‐aligned, and the fracture line became progressively less distinct, nearly disappearing by the 12th week. The hallmark events of fracture healing involve new bone ingrowth and trabecular formation, necessitating the maintenance of a structurally and physiologically stable osteogenic environment.^[^ ^64^ ^]^ The ALAP‐enabled CPA adhesive served a dual function: it securely fixed bone fragments at the fracture site through the robust mechanical and adhesive properties, while also modulating the local immune response and releasing osteogenic substances via multiple bioactivities to expedite the establishment of a favorable osteogenic microenvironment. Importantly, CPA effectively orchestrated the degradation to achieve a concurrent healing process, a stark contrast to CA, which impeded new bone growth.

Micro CT was employed for a comprehensive evaluation of fracture healing. As illustrated in Figure 7C, the CT coronal images provided a clearer depiction of bone cortical interruptions and defects in both the control and CA groups. The 3D reconstructed images highlighted the substantial abnormal growth on the bone surface. Typically, anomalous bone growths were reduced in intensity and incapable of functioning as regular bone tissue. The hyperplasia may result from localized fracture displacement in the control group or pathological stimuli derived from harmful degradation products in the CA group. In contrast, the CPA group showcased the most rapid repair progress. At the 4th week, there was already evident cortical continuity, and by the 8th week, the fracture was essentially healed. 3D reconstruction further revealed that, by the 12th week, the CPA‐repaired bone tissue had completed remodeling, and its morphology closely resembled the normal bone. These findings are consistent with reports on bioactive adhesives incorporating magnesium silicate nanoparticles, which enhance osteoconductivity and accelerate cortical bridging by releasing osteogenic ions (Mg^2^⁺, Si⁴⁺).^[^ ^66^ ^]^

Quantitative Micro CT analysis confirmed the higher bone mineral density (BMD) and new bone content (BV/TV) in the CPA group across all observations (Figure 7D,E). Notably, the proportion of new bone in the CA group was even lower than that in the control group, largely attributed to impaired new bone growth. Similar outcomes were observed in studies comparing calcium phosphate cements (CPCs) and degradable hydrogels, where CPCs exhibited brittle mechanical properties and limited integration with host bone, while hydrogels with dynamic degradation profiles promoted synchronized bone regeneration.^[^ ^64^ ^]^ The hallmark events of fracture healing, including the repair of cortical continuity and recanalization of the marrow cavity, were observed in the CT transverse images at postoperative 8th week, signifying the significant efficacy of CPA in promoting fracture healing and remodeling (Figure 7F). This rapid medullary recanalization mirrors the effects of catechol‐functionalized hydrogels reported by Liu et al., where antioxidant and angiogenic properties facilitated metabolite transport and osteoclast‐osteoblast coupling.^[^ ^67^ ^]^ Effective morphological remodeling was a crucial prerequisite for the restored bone to regain its supportive and locomotor functions. This process relied on the coordinated activity of osteoblasts and osteoclasts in a well‐ordered biological manner. ALAP and caffeic acid collectively enhanced the biological function of CPA, enabling the modulation of the immune status and the swift establishment of a biological microenvironment conducive to the normal functioning of cells and factors. This synergy empowered the CPA group to achieve rapid and efficient bone and medullary cavity remodeling, and the reconnected medullary cavity promotes the transport of metabolites, leading to an active and accelerated remodeling‐repair cycle.

Three‐point bending tests were conducted on radius specimens obtained at the 12th week for biomechanical evaluation (Figure 7G). This approach is consistent with ASTM F2721‐09 standards for evaluating bone‐implant constructs, which emphasize simulating dynamic physiological loads to predict clinical performance.^[^ ^68^ ^]^ The CPA group exhibited superior mechanical properties, with bending strengths closely approaching those of normal bone tissue (Figure 7H). The selection of the three‐point bending test for biomechanical evaluation of in vivo specimens was based on its ability to simulate physiological loading conditions encountered during functional bone use. While lap‐shear and face‐to‐face mechanical tests (Figure 4D–I) were employed in vitro to quantify interfacial adhesion strength under standardized conditions, direct numerical comparisons between these distinct testing modalities are inherently limited by differences in stress distribution and failure mechanisms. For instance, lap‐shear tests predominantly measure interfacial shear strength, whereas three‐point bending evaluates bulk material resilience under flexural stress, as highlighted in biomechanical studies comparing adhesive and screw‐plate fixation systems.^[^ ^69^ ^]^ Nevertheless, the consistent superiority of CPA over control groups across both in vitro adhesion tests and in vivo biomechanical assessments underscores its dual capability to achieve robust interfacial bonding and restore structural integrity under dynamic physiological loads. This multifaceted validation highlights CPA's translational relevance for clinical fracture repair. This reaffirms that the effectively remodeled bone tissue in the CPA group successfully restored the original supportive function. Notably, the CA group displayed the lowest critical fracture strength, indicating that the deficient bone tissue enveloped by peripheral osteophytes severely impairs the functional reconstruction of the fracture. This underscores the importance of not only strong adhesion but also good biocompatibility and controlled degradation for effective bone adhesives.

In consideration of biosafety, blood samples were collected from experimental animals on 7th postoperative day. The results of blood screening indicated no significant abnormalities in the CP and CPA groups, except for mildly elevated inflammatory markers (Figure 7I,J). The transient inflammatory response in the CPA group is consistent with studies on degradable hydrogels, where initial immune activation resolves as the material degrades, enabling a pro‐regenerative microenvironment.^[^ ^70^ ^]^ In contrast, the CA group exhibited more pronounced elevation of inflammatory indexes, suggesting that the implantation of CA triggered a reactive inflammatory response. This observation further elucidated the source of the substantial pathological tissue proliferation observed in the CA group.

**Figure 8:**
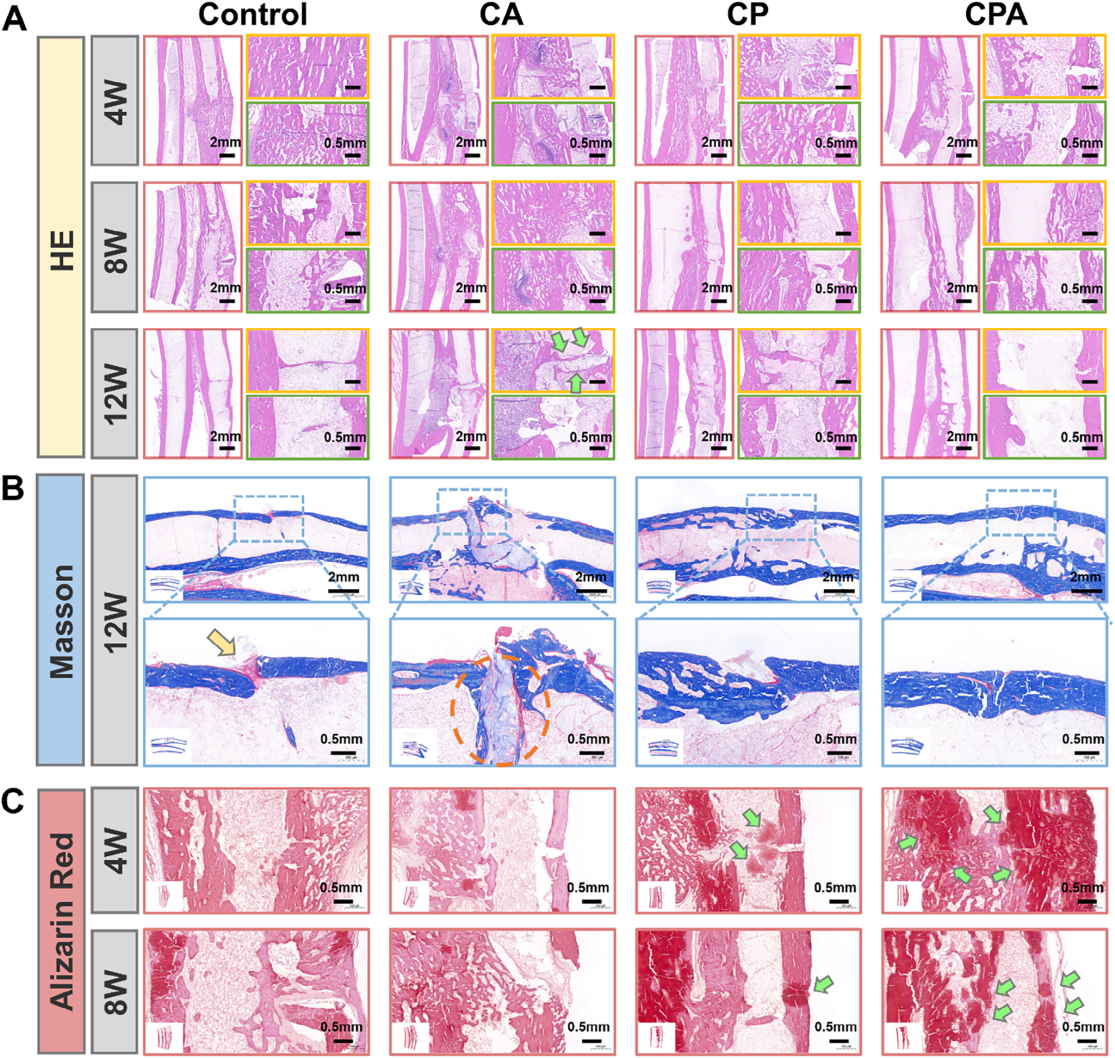
Evaluation of the healing of the radius fracture model on rabbits. A) Histological evaluation through H&E staining at 4, 8, and 12 weeks. B) Representative images of Masson staining in the fracture regions at 12 weeks (yellow arrow indicating bone cortex interruption; orange circle indicating residual CA at the fracture site). C) Histological evaluation through alizarin red staining at 4 and 8 weeks.

Fracture healing was assessed through histological analysis. HE staining indicated delayed cortical remodeling in the control group, evidenced by persistent trabecular disorganization at 12 weeks (**Figure** **8**A). This disorganization, characterized by irregular trabecular alignment and fragmented bone matrix, reflects a detrimental cycle of regeneration‐destruction due to inadequate stabilization. In contrast, the CPA group exhibited well‐organized trabecular structures and cortical continuity, consistent with the superior BV/TV (*p*< 0.001) and BMD (*p*< 0.01) observed in micro‐CT analysis (Figure 7D,E). These outcomes align with bioactive adhesives incorporating magnesium silicate nanoparticles, which promote osteoblast proliferation and collagen alignment through sustained Mg^2^⁺ release, as demonstrated by Gaharwar et al.^[^ ^66^ ^]^

Histological observations indicated evident fracture nonunion in the CA group, characterized by dense bone formation at fragment ends and residual adhesive obstruction. As indicated by the green arrows, the dense bone formation at the ends of the fracture segments entirely occluded the medullary cavity, resulting in the loss of fracture healing potential and the emergence of a pathological pseudoarthrosis. Masson staining at the 12th week provided a clearer view of the final healing outcomes (Figure 8B). In the control group, cortical continuity remained unestablished at the 12th week (highlighted by the yellow arrow), signifying a notable delay in the fracture healing process. Importantly, non‐degradable CA was detected at the fracture site (indicated by orange circles). CA filled the gap between the fracture segments, creating an impediment to bone growth. Dense bone formation occurred on both sides of the CA, indicating the onset of fracture nonunion. Such outcomes contrast sharply with degradable hydrogels like CPA, which facilitate synchronized degradation and osteogenesis, as shown in Xing et al.’s work on diabetic fracture healing.^[^ ^71^ ^]^

In the CPA group, fracture reconstruction was successfully achieved by the 12th week, resulting in the formation of a regular bone cortex. Conversely, while a healing trend was apparent in the CP group, the outer cortex remained incompletely closed, indicating an ongoing healing process. The accelerated cortical bridging in the CPA group may stem from its dual functionality: ALAP‐mediated immunomodulation (via Mg^2^⁺ release) and caffeic acid's antioxidant activity, which collectively suppress inflammation and enhance osteoblast differentiation.^[^ ^67^ ^]^ Alizarin red staining was conducted at the 4th and 8th weeks to assess osteogenesis (Figure 8C). The CPA group exhibited dense, interconnected calcified nodules at 8 weeks (indicated by arrows in Figure 8C), characteristic of early lamellar bone formation, as supported by micro‐CT analysis showing cortical continuity and elevated BMD (Figure 7D,E). These findings underscored the reliable adhesion ability and osteogenic induction properties of CPA, demonstrating its capacity for achieving stable reconstruction and accelerating the healing process in rabbit radius fractures.

Compared to current bone adhesives and fixation approaches, CPA demonstrates multifaceted superiority: 1) Cyanoacrylate (CA) Adhesives: While CA exhibits strong adhesion (≈166.1 kPa), its brittleness, non‐degradability, and cytotoxicity hinder clinical utility.^[^ ^64^ ^]^ In contrast, CPA achieves comparable adhesion strength (152.1 kPa) while integrating biodegradability and osteogenic activity, addressing CA's limitations. 2) Calcium Phosphate Cements: CPCs are osteoconductive but lack adhesive strength and flexibility.^[^ ^72^ ^]^ CPA's dual biomimetic design merges adhesion and osteoinduction, enabling simultaneous fixation and healing. 3) Bioactive Glasses: These materials promote bone regeneration but suffer from poor mechanical strength.^[^ ^73^ ^]^ CPA's organic–inorganic network achieves compressive strength (≈14.5 MPa) comparable to trabecular bone, overcoming this drawback. CPA's key advantages can be summarized as below: 1) Dual Biomimetic Design: Structural reinforcement (collagen/LAP) mimics bone's organic–inorganic matrix, while catechol‐mediated adhesion and antioxidant activity replicate mussel‐inspired wet adhesion.^[^ ^74^ ^]^ 2) Controlled Degradation: CPA degrades within 5–6 weeks, aligning with fracture healing timelines, whereas non‐degradable adhesives (e.g., CA) impede remodeling.^[^ ^65^ ^]^ 3) Immunomodulation: CPA promotes M2 macrophage polarization, resolving inflammation and accelerating osteogenesis—a feature absent in traditional adhesives.^[^ ^57^, ^62^, ^75^ ^]^

## 3 Conclusion

In summary, we have successfully developed a mechanically enhanced bone adhesive that effectively integrates the essential criteria of reliable adhesive strength and osteogenic activity. Leveraging organic–inorganic and multiple cross‐linking design, ALAP can be evenly distributed within the hydrogel network, serving as a physical reinforcement and biological functions, to guide subsequent fracture healing. The adhesive's robust mechanical and adhesive properties enabled secure bonding of comminuted fragments from porcine femur or rabbit radius, facilitating the anatomical repositioning even in blood‐rich environments. In a pioneering evaluation using a comminuted radius fracture model in rabbits, this proposed adhesive consistently stabilized fracture fragments and accelerated fracture healing by swiftly establishing a physically and biologically osteogenic microenvironment. This meticulously designed bone tissue adhesive is anticipated to offer innovative solutions and protocols for the clinical management of complex fractures.

## 4 Experimental Section

### Materials

Collagen (Mw≈60 kDa) and β‐alanine standards were purchased from Sigma–Aldrich. 1‐(3‐Dimethylaminopropyl) −3‐ethylcarbodiimide hydrochloride (EDCI) and NHS were purchased from J&K Scientific. Caffeic acid (Mw≈180.16 Da), ethylenediamine, dimethyl sulfoxide (DMSO), and ethylene glycol were purchased from ENERGY CHEMICAL. LAP was supplied by BYK Additives & Instruments. Tetra‐PEG‐SC was obtained from JenKem Technology Co., Ltd. Fibrin glue was purchased from Guangzhou Beixiu Bio‐Technology Co., Ltd. EDTA was purchased from Tianjin Zhiyuan Chemical Reagent Co., Ltd. (3‐Aminopropyl) triethoxysilane and n‐hexane were purchased from Aladdin Biochemical Technology Co., Ltd.

### Preparation of CAC

A total of 5 g of Col was dissolved in 100 mL of PBS, followed by the addition of 16 mL of ethylenediamine, and pH adjustment to 5.0 with dilute hydrochloric acid. After returning to room temperature, 2.5 g of EDCI was added, and the reaction proceeded for 12 h. The resulting product was subjected to a 3‐day dialysis process and subsequently freeze‐dried to obtain AC. 1 g of AC was dissolved in a 50 mL mixture of deionized water and DMSO (molar ratio 5:2) at pH 5.0. Additionally, 0.01 g of caffeic acid, 0.25 g of EDCI, and 0.15 g of NHS were dissolved in 10 mL of DMSO. This mixture was added to the AC solution, adjusting the pH to 4.5, and the reaction occurred over 24 h. After an initial 12 h reaction period, the solution was returned to room temperature. After the introduction of 2.5 g of EDCI, the reaction continued for an additional 24 h under nitrogen protection. The resulting reaction product underwent dialysis in acidified deionized water (pH = 5.0) for 3 d, followed by dialysis in neutral deionized water (pH = 7.0) for 4 h. Finally, the material was freeze‐dried to yield CAC. Primary amino groups in collagen (Col) and modified AC were quantified via the TNBS assay. Solutions of Col and AC (0.5 mol L^−1^ in PBS, pH 7.4) were mixed with 4 wt.% NaHCO₃ and 0.1 wt.% TNBS (1:1:1 v/v), incubated at 50 °C for 2 h in the dark, and absorbance was measured at 415 nm. A linear calibration curve (y = 1.45x+0.33, R^2^ = 0.997) was generated using β‐alanine standards (0.1–1.0 mmol L^−1^). The grafting degree of caffeic acid (4.5%) was calculated with a 1H NMR spectrum by comparing the integral ratios of caffeic acid aromatic protons (δ 6.5–7.2 ppm) to collagen backbone protons (δ 1.0–4.5 ppm).

### Preparation of ALAP

In a double‐necked flask, 1.6 g of LAP was combined with 100 mL of n‐hexane. Subsequently, 25 mL of (3‐aminopropyl) triethoxysilane were added with magnetic stirring. The reaction was conducted under nitrogen protection in a water bath at 60 °C for 24 h. The resulting product was subjected to three washes with ethanol and then dried in an oven to obtain ALAP.

### Preparation of CP, CPL, and CPA

The CAC and tetra‐PEG‐SC solutions, prepared as previously described, were dissolved in PBS. Equal volumes of these two solutions were combined and thoroughly mixed through agitation to create a CP hydrogel. To form CAC‐LAP and CAC‐ALAP solutions, LAP and ALAP were added proportionally at concentrations ranging from 1% to 3% (w/v) to the CAC solution. Subsequently, these CAC‐LAP and CAC‐ALAP solutions were mixed with an equal volume of the tetra‐PEG‐SC solution, resulting in the preparation of CPL and CPA hydrogels.

### Characterization

^1^H NMR (Bruker Ascend 400 MHz, Switzerland) patterns were obtained with a nuclear magnetic resonance spectrometer (Bruker Ascend 400 MHz, Switzerland). FT‐IR spectroscopy was conducted using a TENSOR‐27 spectrometer (Bruker, Germany) with settings that included a scanning frequency range of 4000–400 cm^−1^, a resolution of 2 cm^−1^, and an average of 32 scans.

Powdered LAP/ALAP samples underwent grinding and drying were observed with TEM (Tecnai 20 200kV, FEI, American) to measure their particle size. XRD was performed with an X‐ray diffractometer (Rigaku SmartLab SE, Japan) and the measurements covered a 2θ range of 5°–80°, with a scanning speed of 2 min^−1^. The light source was Cu‐Kα rays, and the tube voltage and current were 40 kV and 40 mA, respectively. The microstructure of the samples was observed with SEM (JSM‐6700F, JEOL, Japan) at a voltage of 10 kV. EDS spectra and elemental maps were acquired in mapping mode (1024 × 884 pixels, 50 µs dwell time). AFM (Bruker Dimension Icon, Germany) was utilized to observe the surface morphology and analyze the topography of the samples. Representative images were saved for further analysis.

Elemental analysis was performed using a Vario EL Cube CHNS‐O analyzer (Elementar, Germany). Samples (5–10 mg) were combusted at 1150 °C under oxygen flow. Nitrogen content was quantified via thermal conductivity detection. Calibration was performed using sulfanilamide as a standard. Three independent measurements were conducted for each sample, and results were normalized to dry weight.

### Injectability

The CAC/ALAP solution, pre‐dyed in blue, was mixed with an equal volume of tetra‐PEG‐SC solution. This mixed solution was then drawn into a syringe. As the solution approached the gel state, it was injected into a beaker filled with water using a 22 G needle. The resulting morphology of the injected solution was observed.

### Gelation time

The gelation time was determined through a tube tilting experiment. In this procedure, 200 µL of CAC, CAC/LAP, and CAC/ALAP solutions were added to a sample vial that already contained 200 µL of tetra‐PEG‐SC solution. The solutions were thoroughly mixed and agitated. Gelation was deemed to occur when the solution no longer flowed upon tilting the sample vial, at which point the gelation time was recorded.

### Rheology

Rheology tests were performed on a rheometer (Thermo Haake, American) with a 35 mm‐diameter conical plate. The test temperature was 25 °C, and the plate gap was 0.05 mm. Dynamic strain sweeps were performed at a fixed frequency of 1.0 rad s^−1^ with strains ranging from 0.05% to 100%. Dynamic frequency sweeps were performed at a fixed strain of 0.05% with a sweep frequency range from 0.1 to 10 rad s^−1^.

### Swelling and Degradation

The initial weight (W_0_) of the prepared hydrogels was weighed, then the hydrogels were placed in a sample bottle containing an excess of PBS and shaken at 25 °C at a constant speed. The samples were removed at regular intervals and weighed after removing excess water.

The swelling ratio (SR) was calculated as:

(1) $$\mathrm{SR}\left(\%\right)=\left(W_{t}-W_{0}\right)/W_{0}\times 100\%$$

The degradation ratio (DR) was calculated as:

(2) $$\mathrm{DR}\left(\%\right)=\left(W_{0}-W_{t}\right)/W_{0}\times 100\%$$

W_0_ and W_t_ respectively, signify the initial weight and weight at various observation times. The test was repeated 3 times.

### Mechanical Properties

The hydrogels were fabricated in cylindrical and bar shapes to facilitate compression and tensile testing. Compressive and tensile strengths were assessed using a universal tensile machine (3365 Instron, USA). For compression testing, a speed of 2 mm min^−1^ was employed, with a compressive strain set at 97% and a maximum load of 4000 N. Tensile testing was conducted at a rate of 50 mm min^−1^. Three separate tests were performed for each sample.

### Cyclic Compression Testing

The CPA hydrogel (10 mm in diameter and 5 mm in height) was placed in a universal testing machine (3365 Instron) and subjected to 25 cycles of cyclic compression at a rate of 1 mm/min^−1^ (strain range: 0–60%). Stress–strain curves were recorded, and hysteresis energy loss was calculated as the area difference between the nth cycle and the first cycle curves. Each group was tested 5 times, with data expressed as mean ± standard deviation. Testing was conducted in a 37 °C PBS solution to simulate physiological conditions.

### Post‐Swelling Mechanical and Adhesive Testing

To evaluate the mechanical stability of CPA under physiological conditions, fully cured CPA hydrogels and CPA‐bonded porcine bone fragments were immersed in PBS at 37 °C for 24 h. Mechanical tests (compression and lap‐shear adhesion) were performed immediately after removing excess surface fluid. Compressive strength was measured using a universal testing machine (Instron 3365) at a strain rate of 2 mm min^−1^. Adhesive stress was quantified via lap‐shear testing (10 mm min^−1^) on fresh porcine bone substrates. Five independent replicates were conducted for each condition.

### Mechanical and Adhesive Testing of EDTA‐Treated CPA Hydrogels

To investigate the role of Mg^2^⁺‐catechol coordination in the hydrogel network, ALAP nanoparticles were first treated with EDTA (pH 7.4) for 24 h to chelate and remove surface Mg^2^⁺ ions, followed by thorough rinsing with deionized water and lyophilization. CPA‐EDTA hydrogels were synthesized using EDTA‐treated ALAP under identical fabrication conditions as standard CPA. For compressive mechanical testing, cylindrical CPA‐EDTA samples (10 mm diameter × 5 mm height) were subjected to uniaxial compression at a strain rate of 2 mm min^−1^ using a universal testing machine (Instron 3365). Adhesive performance was evaluated via lap‐shear testing on fresh porcine bone substrates (10 mm min^−1^ displacement rate). To ensure physiological relevance, all tests were conducted in a 37 °C PBS environment. Five independent replicates were performed for each group.

### Adhesion Test

Fresh porcine scapulae and femurs were sectioned into irregular pieces, with subsequent reassembly with CPA. Observations for axial and tangential loads were conducted at 1 and 15 min of resting, respectively. Following a 12 h resting period, the maximum load was tested, and the resulting maximum load value was recorded using an electronic scale. Furthermore, Strips of porcine bone, measuring 40 mm × 20 mm × 10 mm, were bonded using hydrogels and cyanoacrylate (CA) through both lap‐shear and face‐to‐face methods. After a 2 h resting at room temperature, mechanical testing of the bonded fragments was conducted with a universal tensile machine at a rate of 10 mm min^−1^. Additionally, the segmented porcine femur was reconstructed and fixed in vitro using internal fixation instruments and CPA in accordance with the AO standard for internal fixation of comminuted distal femur fractures. Imaging assessments were conducted using X‐ray (Cheetah EVO, Germany) and CT scans (SIEMENS, Germany).

### Cytocompatibility Test

NIH‐3T3 cells were cultivated in Dulbecco's Modified Eagle Medium (DMEM, Gibco, Thermo Fisher Scientific, USA) and seeded into 24‐well plates. Hydrogels were fully cured before cell seeding. Specifically, the hydrogels (CP, CPL, CPA) were allowed to fully cure at room temperature (gelation time as shown in Figure 2C of the main text) before being placed in 24‐well plates. The cured hydrogels were washed three times with PBS to remove unreacted substances. Subsequently, cell suspensions (density: 1 × 10 ^ 4 cells/well) were directly seeded onto the hydrogel surfaces to avoid physical interference from uncured gel during cell culture. Hydrogel Extract Preparation: Hydrogels were immersed in serum‐free DMEM at a ratio of 1:10 (w/v, hydrogel to medium) and incubated for 24 h at 37 °C. The mixture was centrifuged at 3000 rpm for 10 min, and the supernatant was sterile‐filtered through a 0.22 µm membrane. The pre‐prepared hydrogel extract was introduced into the well plates and co‐incubated with the cells at 37 °C. Cell proliferation and viability were evaluated on days 1, 2, and 3 using the CCK‐8 kit (Dojindo Molecular Technologies, Japan) and live/dead cell double staining kit (Thermo Fisher Scientific, USA), with complete medium (DMEM containing 10% fetal bovine serum (FBS, Gibco, Thermo Fisher Scientific, USA) and 1% penicillin/streptomycin, Sigma–Aldrich, USA) serving as the control.

The cell viability was calculated:

(3) $$\begin{array}{cc} \mathrm{Cell}\;\mathrm{viability}\left(\%\right) & =\left(OD_{\mathrm{sample}}-OD_{\mathrm{blank}}\right)/\left(OD_{\mathrm{control}}-OD_{\mathrm{blank}}\right)\\ & \;\times 100\% \end{array}$$

OD_sample_ denotes the OD values of the hydrogel groups, while OD_blank_ and OD_control_ represent the OD values of the blank medium and the control group on day 1, respectively.

### Hemolysis Test

Blood from SD rats, anticoagulated with heparin, was centrifuged to separate the precipitated red blood cells (RBCs). Following three washes with PBS, the RBCs were diluted to a 5% (v/v) concentration in saline. Subsequently, 100 µL of hydrogel (hydrogels were fully cured), 0.9% saline, and 0.1% Triton X‐100 were dispensed into separate centrifuge tubes, each containing 500 µL of the diluted RBCs. The tubes were then incubated in a shaking incubator at 37 °C. Saline and Triton X‐100 were set as negative and positive controls, respectively. After 12 h of incubation, the mixture was centrifuged for 10 min to obtain the supernatant, and the absorbance was measured at 540 nm.

The hemolysis ratio was calculated as:

(4) $$\begin{array}{cc} \mathrm{Hemolysis}\left(\%\right) & =\left(OD_{\mathrm{sample}}-OD_{\mathrm{negative}}\right)/\left(OD_{\mathrm{positive}}-OD_{\mathrm{negative}}\right)\\ & \;\times 100\% \end{array}$$

OD_sample_ represents the OD values of the hydrogel groups, while OD_negative_ and OD_positive_ denote the OD values of the blank and Triton X‐100 groups, respectively.

### Antioxidant Properties

The fully cured hydrogels were pulverized into a homogenate and then dispersed in ethanol at a concentration of 2 mg mL^−1^. In parallel, an ethanol solution of vitamin C at the same concentration served as the positive control. Following this, 100 µM DPPH was introduced to each of the prepared samples, and the mixtures were incubated for 30 min in a light‐protected environment. The 1,1‐diphenyl‐2‐pyridohydrazino (DPPH) absorption wavelength for each sample was measured and recorded.

The DPPH scavenging was calculated as:

(5) $$\mathrm{DPPH}\;\mathrm{scavenging}\left(\%\right)=\left(A_{B}-A_{N}\right)/A_{B}\times 100\%$$

A_B_ represents the wavelength of the blank group, while A_N_ represents the wavelength of both the hydrogel and the vitamin C group.

### Angiogenesis Experiment

A 100 µL suspension of human umbilical vein endothelial cells (HUVEC) was seeded into a 96‐well plate at a density of 1 × 10^5^ cells mL^−1^. Subsequently, 100 ng mL^−1^ of vascular endothelial growth factor (VEGF), along with the hydrogels’ immersion solution, was added. After a 6 h incubation, the total length of tubular structures in randomly selected fields was calculated. This process was repeated three times for each group. For supernatant experiments, hydrogels were incubated in cell culture medium (1:1 w/v) for 24 h, centrifuged, and sterile‐filtered. Detailed Supernatant Preparation Protocol: 1) Hydrogel Incubation: Fully cured hydrogels (weight‐to‐volume ratio of 1:1, e.g., 100 mg hydrogel in 100 µL medium) were immersed in DMEM supplemented with 10% fetal bovine serum (FBS) and incubated at 37 °C under 5% CO₂ for 24 h. 2) Centrifugation and Filtration: The hydrogel‐medium mixture was centrifuged at 3000 rpm for 10 min. The supernatant was then sterile‐filtered through a 0.22 µm membrane to remove residual gel fragments or nanoparticles. 3) Application: The filtered supernatant was directly applied to HUVEC (human umbilical vein endothelial cell) cultures for angiogenesis assays, ensuring conditions mimicking the in vivo microenvironment.

### Transwell Assay

The hydrogels were placed in a 24‐well plate, and single‐cell suspensions of trypsin‐treated HUVEC at the logarithmic growth stage were prepared. In the Transwell system, 600 µL of low‐serum medium was added to the lower chamber, and 100 µL of single‐cell suspension from each group was added to the upper chamber. Cell Preparation: HUVEC cells were cultured in DMEM supplemented with 10% FBS until reaching 70–80% confluence (logarithmic growth phase). Cells were detached using 0.25% trypsin‐EDTA for 3 min at 37 °C, neutralized with complete medium, centrifuged at 1000 rpm for 5 min, and resuspended in serum‐free DMEM to obtain single‐cell suspensions. Experimental Groups: Groups included: 1) Control (cells without hydrogel exposure), 2) CP, 3) CPL, and 4) CPA. Transwell Setup: Hydrogels (CP, CPL, CPA) were placed in the lower chamber of a 24‐well plate. The lower chamber was filled with 600 µL of low‐serum medium (DMEM with 2% FBS). 100 µL of single‐cell suspension (1 × 10⁵ cells mL^−1^) from each group (control, CP, CPL, CPA) was added to the upper chamber. After 48 h of incubation, the culture medium was discarded, non‐migrated cells were gently removed, and the remaining cells were fixed with paraformaldehyde and stained with crystal violet. Random fields of view were selected, photographed under a microscope, and subjected to cell counting.

### Osteogenic Activities

BMSCs were isolated from the bone marrow of New Zealand white rabbits (age: 12–14 weeks, weight: 2.5–3 kg, purchased from Vital River Laboratories, Beijing, China) used in the study. BMSCs were cultured in DMEM supplemented with 10% FBS and 1% penicillin/streptomycin. Cells were seeded at a density of 1 × 10⁵ cells mL^−1^ and cultured with fully cured hydrogels under standard conditions (37 °C, 5% CO₂). For osteogenic differentiation, BMSCs were induced with osteogenic medium (DMEM containing 10% FBS, 50 µg mL^−1^ ascorbic acid (Sigma–Aldrich, USA), 10 mM β‐glycerophosphate (Sigma–Aldrich, USA), and 100 nM dexamethasone (Sigma–Aldrich, USA)) for 14 days. Subsequently, ALP and calcium nodule staining were performed using an ALP staining kit (Beyotime, Wuhan, China) and an alizarin red staining kit (Tiandz, Beijing, China), respectively. Quantification of ALP activity was performed using an ALP quantification kit (Jiancheng, Nanjing, China), and calcium levels were assessed using cetylpyridinium chloride (TCI, Shanghai, China) in conjunction with a spectrophotometer set at 570 nm for measurement.

### Bioinformatic Analysis

BMSCs were co‐cultured with hydrogel extracts (1:10 w/v, hydrogel‐to‐medium ratio) for 48 h under standard conditions (37 °C, 5% CO₂). Total RNA was extracted using TRIzol reagent (Invitrogen, USA), and cDNA synthesis was performed with PrimeScript RT Master Mix (Takara, Japan). RNA sequencing libraries were prepared using the NEBNext Ultra II RNA Library Prep Kit (NEB, USA) and sequenced on an Illumina NovaSeq 6000 platform (150 bp paired‐end reads). Raw reads were aligned to the reference genome (Oryctolagus cuniculus OryCun2.0) using the STAR aligner, and read counts were normalized using DESeq2. DEGs were defined as genes with |log₂(fold change)| > 1 and adjusted *p*‐value< 0.05 after Benjamini–Hochberg false discovery rate correction. Raw sequencing data were deposited in the GEO database. Raw sequencing data were deposited in the GEO database. RNA sequencing data were analyzed using the DAVID 2021 database for GO and KEGG pathway enrichment. For GO analysis, all DEGs were pooled, and significantly enriched terms in biological processes, cellular components, and molecular functions were identified. Heatmaps and volcano plots (DEGs meeting the thresholds were highlighted) were generated using RStudio (v4.2.1, Posit Software, USA) with the pheatmap and EnhancedVolcano packages.

### In Vitro Macrophage Polarization Assays

BMDMs were isolated from 8‐week‐old female C57BL/6 mice (Vital River Laboratories, Beijing, China). Bone marrow cells were flushed from femurs and tibiae, cultured in DMEM supplemented with 10% FBS and 20 ng mL^−1^ M‐CSF for 7 days to induce macrophage differentiation. For qRT‐PCR, total RNA was extracted using TRIzol, and gene expression of M1 (CD68) and M2 (CD206, CD31) markers was quantified using SYBR Green Master Mix (Roche, Switzerland) on a QuantStudio 5 system (Thermo Fisher, USA). Primer sequences for CD68 (F: 5′‐CTGCTGTCTCAATGAGGACAC‐3′, R: 5′‐TCTGGATCATCAGCACAGGT‐3′), CD31 (F: 5′‐CCTGGTGCTGTTTCTCTGCT‐3′, R: 5′‐GCCATCCAGGTGTTCTCCAT‐3′), and CD206 (F: 5′‐CAGGTGTGGGCTCAGGTATT‐3′, R: 5′‐CGGTTGGAACACCTCGTAGA‐3′) were designed using NCBI Primer‐BLAST. For immunofluorescence, BMDMs were fixed with 4% paraformaldehyde, permeabilized with 0.1% Triton X‐100, and incubated with anti‐CD68 (Abcam ab213363, 1:200), anti‐CD206 (CST #24595, 1:100), and anti‐CD31 (Santa Cruz sc‐376764, 1:50) antibodies. Alexa Fluor 488/594‐conjugated secondary antibodies (Invitrogen, 1:500) and DAPI nuclear staining were used. Relative gene expression was calculated using the ΔΔCt method, normalized to GAPDH. Fluorescence Microscopy images were acquired using an EVOS FL Auto Imaging System (Thermo Fisher Scientific, USA).

### Rabbit Radius Comminuted Fracture Model

A total of 48 female rabbits (12 per group, 4 groups: control, CA, CP, CPA), weighing between 2.5 and 3 kg, were chosen and anesthetized with 3% pentobarbital. At postoperative weeks 4, 8, and 12, 3 rabbits were euthanized from each group for imaging (X‐ray and micro‐CT) and histological analysis (H&E, Masson, alizarin red staining). An additional 3 rabbits were euthanized for biomechanical testing (three‐point bending) at week 12 after surgery. Subsequently, the left forelimb was shaved, and iodophor was applied for disinfection. A longitudinal incision, ≈2 cm in length, was then made along the radial side of the forearm to expose the radius. To establish a standardized and consistent comminuted fracture model, two incisions were made in the anterior middle third region of the radius, creating a segmental bone defect ≈10 mm in length. With a saw blade thickness of 1.5 mm, the resulting length of the truncated bone fragments was ≈7 mm (10 mm–1.5 mm × 2). The fracture fragments could not be immobilized in their original positions through simple resetting. Subsequently, the bone adhesives, as well as commercially available CA, were employed for adhesion to repair the fracture. The blank control group was to reposition the bone fragments directly without any special treatment. Following effective hemostasis, the incision was closed in layers and sterilized. In this context, the experimental animals were categorized into four groups: the control group, the CA group, the CP group, and the CPA group. The animals were euthanized at 4, 8, and 12 weeks after surgery, and the ulnar radius was extracted for imaging, biomechanical, and histological assessments. The experimental animals were sourced from the Experimental Animal Center of PLA General Hospital. All animal handling procedures were conducted in compliance with the approved protocol by the Ethics Committee of PLA General Hospital.

### Imaging Assessment

Orthopantomograms of the ulnar radius specimens were captured using an X‐ray machine (Cheetah EVO, Germany). Micro‐CT scans were performed with an Inveon MM CT machine (SIEMENS, Germany), and the data were analyzed and reconstructed with Inveon Research Workplace (SIEMENS, Germany) and COBRA Exxim (EXXIM Computing, CA) software. Following the delineation of the region of interest (ROI), the bone volume (BV, mm^3^) and total volume (TV, mm^3^) within the ROI were determined. The new bone content (BV/TV, %) was then calculated, and the bone mineral density (BMD, mg cm^−3^) within the ROI was quantified.

### Biomechanical Assessment

At the 12th postoperative week, specimens from the rabbit ulnar‐radial complex were obtained and subjected to three‐point bending (Instron 3365 Universal Testing Machine, Instron, USA) mechanical tests. These tests were conducted using a universal tensile machine to record critical stresses and breaking loads. Three‐point bending was performed at 1 mm min^−1^ with a 20 mm span. Maximum load and displacement were recorded.

### Blood Test Indicators

The venous blood collected from the experimental rabbits was utilized to measure the following indicators: RBC (red blood cells), HB (hemoglobin), HCT (hematocrit), PCV (packed cell volume), MCV (mean corpuscular volume), MCH (mean corpuscular hemoglobin), MCHC (mean corpuscular hemoglobin concentration), PLT (platelet count), MPV (mean platelet volume), PCT (plateletcrit), WBC (white blood cells), MOS (monocytes), NEU% (neutrophils), LY% (lymphocytes), EOS% (eosinophils), BASO% (basophils), ALT (alanine aminotransferase), AST (aspartate aminotransferase), LDH (lactate dehydrogenase), GGT (gamma‐geranyl transpeptidase), CK (creatine kinase), TBIL (total bilirubin), AMYL (amylase), BUN (blood urea nitrogen), CREA (creatinine), ALB (albumin), GLOB (globulin), A/G (albumin globulin ratio), Ca (calcium), P (phosphonium), K (potassium) Cl (chlorine). ALT, AST, BUN, and CREA assays were performed using kits from Nanjing Jiancheng Bioengineering Institute (Nanjing, China).

### Histological Assessment

Ulnar radius specimens preserved in 10% formalin were embedded and processed as undecalcified hard sections to retain calcium deposits for histological analysis. Sections were prepared using a diamond saw and polished to ensure structural integrity. Histological examinations included HE staining, Masson staining, and Alizarin red staining to evaluate tissue morphology, collagen deposition, and calcium mineralization, respectively.

### Statistical Analysis

All quantified data were reported as mean ± standard deviation. Statistical comparisons were performed using one‐way or two‐way ANOVA along with Tukey‘s multiple comparison test, as well as unpaired two‐tailed *t*‐tests. The analyses were carried out using Origin 9.1 (USA) or GraphPad Prism 7.0 software (USA). Statistical significance was defined as a probability value (P) of less than 0.05 (*p*< 0.05).

### Ethics approval statement

The animal studies were carried out in accordance with the Committee of Chinese PLA General Hospital, IACUC of PLAGH on the use of Institutional Animal Care and Use (Ethics Approval No. SQ2022439).

## Conflict of Interest

The authors declare no conflict of interest.

## Supporting information



Supporting Information

Supplemental Movie 1

Supplemental Movie 2

Supplemental Movie 3

## Data Availability

The data that support the findings of this study are available from the corresponding author upon reasonable request.
