# Supplementary material for: Dual‐Biomimetic Bone Adhesive with Osteoimmunomodulatory Capabilities for Anatomical Reconstruction of Comminuted Fractures
Source: Adv Sci (Weinh). 2025 Jul 8;12(32):e01108. doi: 10.1002/advs.202501108 (PMC12407313; doi:10.1002/advs.202501108)
Supplement: Supplementary file 1 — Supporting Information [file ADVS-12-e01108-s004.docx]

**Supporting Information**

**Dual-Biomimetic Bone Adhesive with Osteoimmunomodulatory Capabilities for Anatomical Reconstruction of Comminuted Fractures**

Junyao Cheng, Hufei Wang, Ming Li, Jianpeng Gao, Xiao Liu, Chuyue Zhang, Pengfei Chi, Bo Li, Yuan Xve, Yifan Wang, Daoyang Fan, Zheng Wang*, Jianheng Liu*, Xing Wang*, and Licheng Zhang*

**Supporting Results**


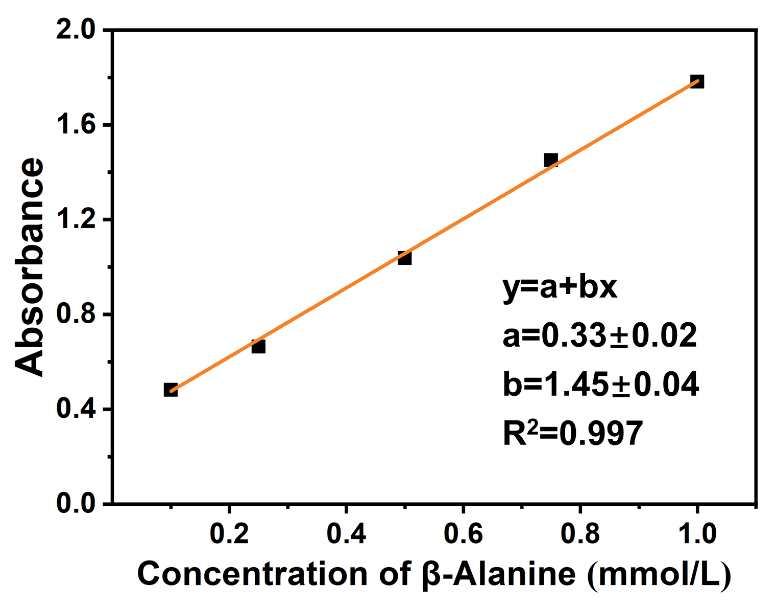


**Figure S1.** Standard curve for quantification of primary amino content by TNBS method.


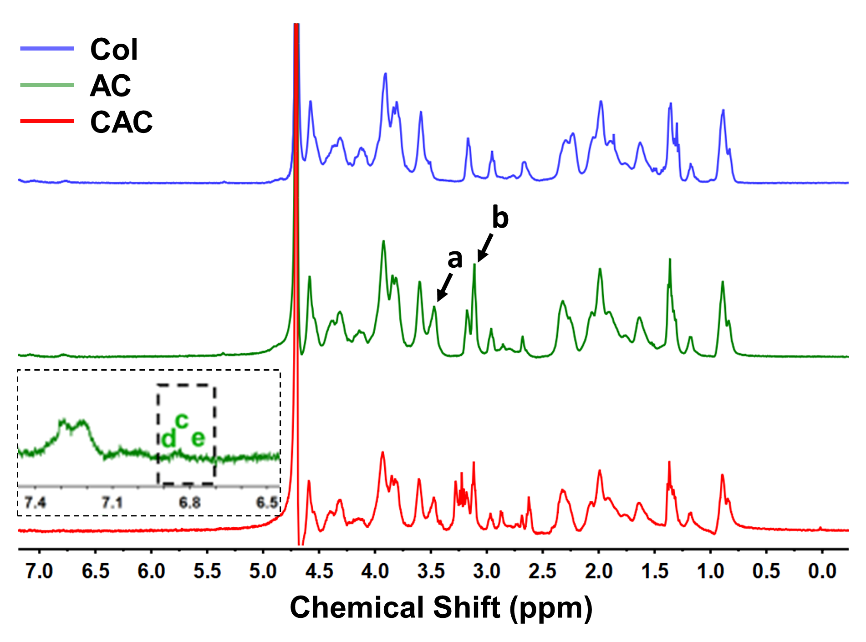


**Figure S2.** ^1^H NMR spectra of Col, AC and CAC.


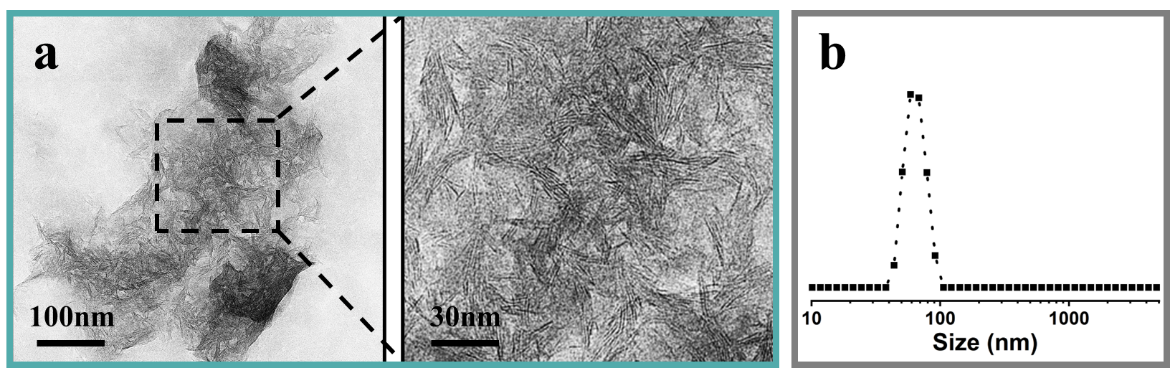


**Figure S3.** TEM images of LAP (a) and particle size analysis curves (b).


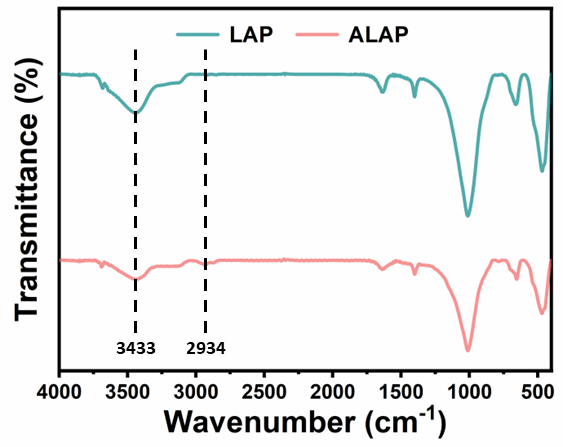


**Figure S4.** FT-IR spectra of LAP and ALAP.


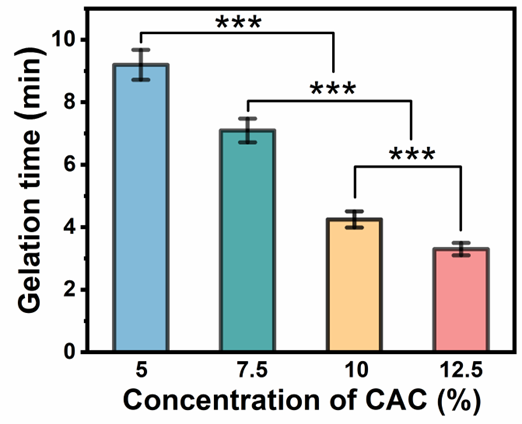


**Figure S5.** Gelation time of CP with different CAC concentrations.


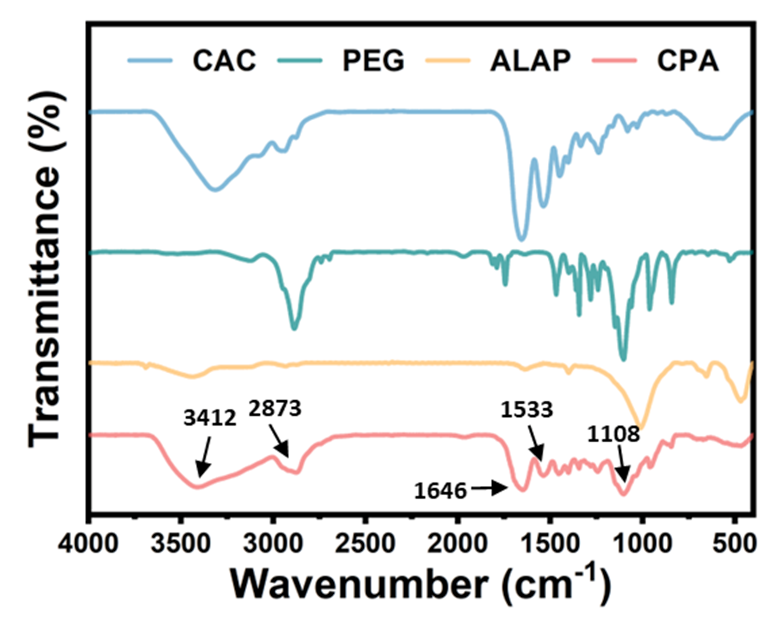


**Figure S6.** FT-IR spectra of Col, AC, and CAC.


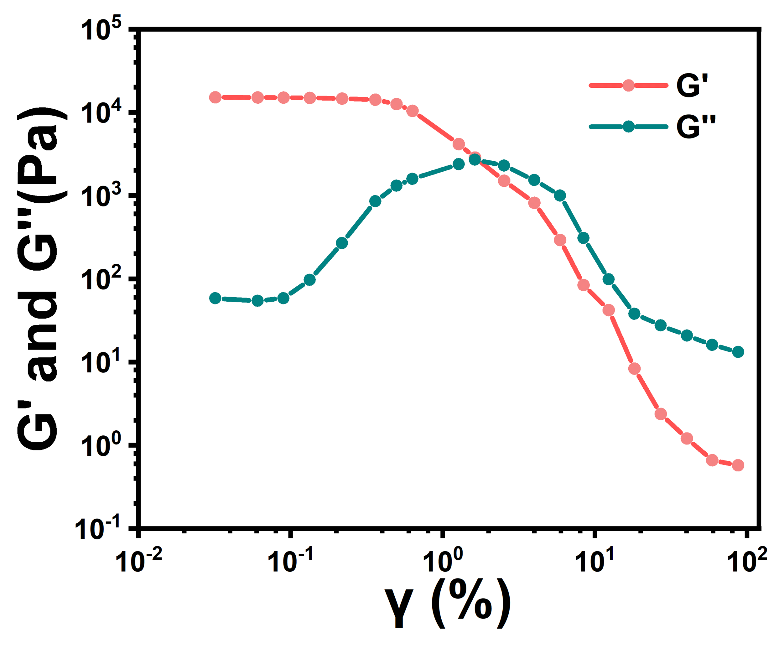


**Figure S7.** Strain sweeps of CPA with an angular frequency of 10 rad/s.


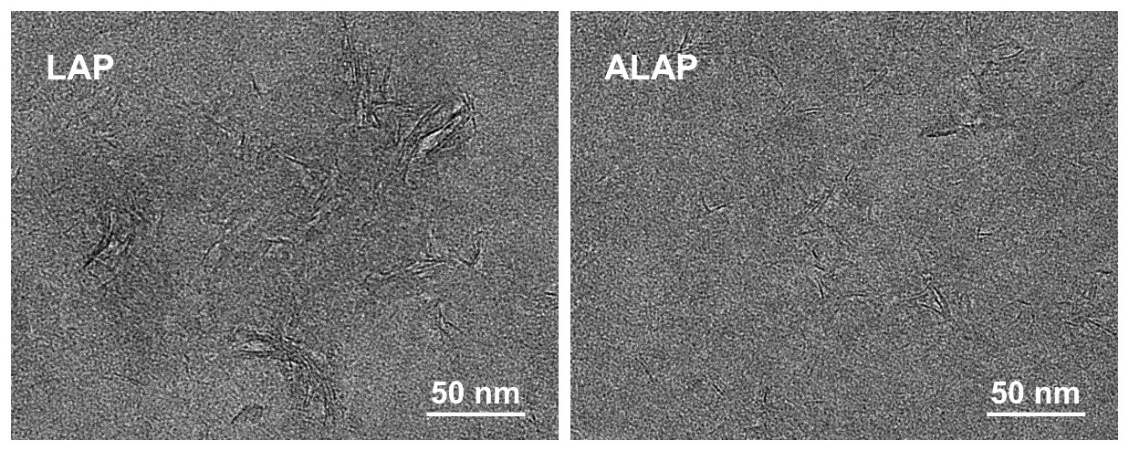


**Figure S8.** TEM images of LAP and ALAP nanoparticles.


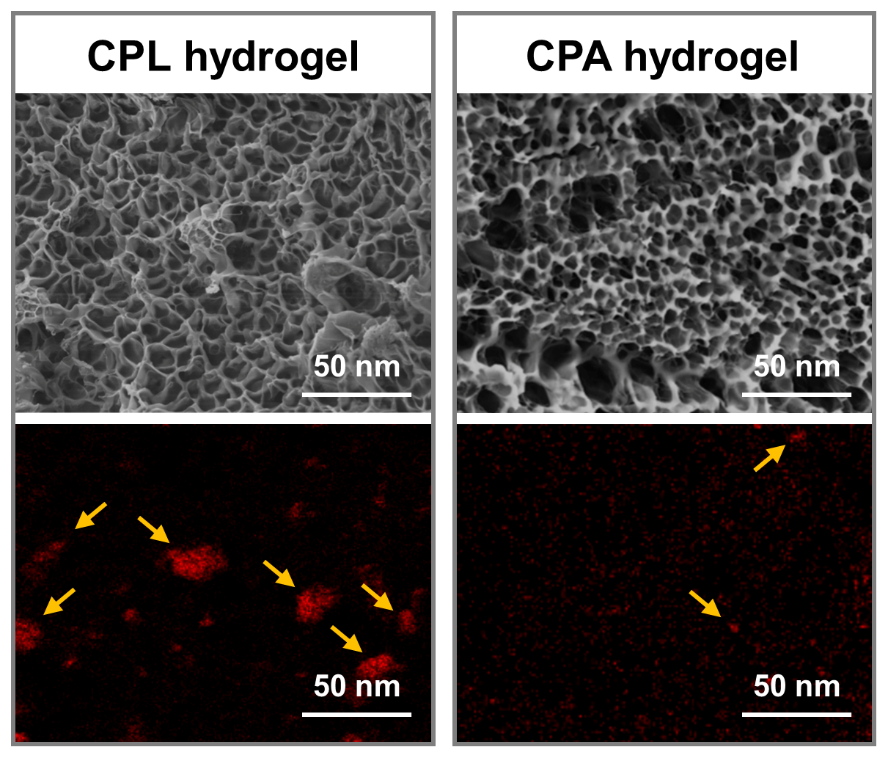


**Figure S9.** EDS images of CPL and CPA hydrogels (yellow arrows displayed localized magnesium clusters).


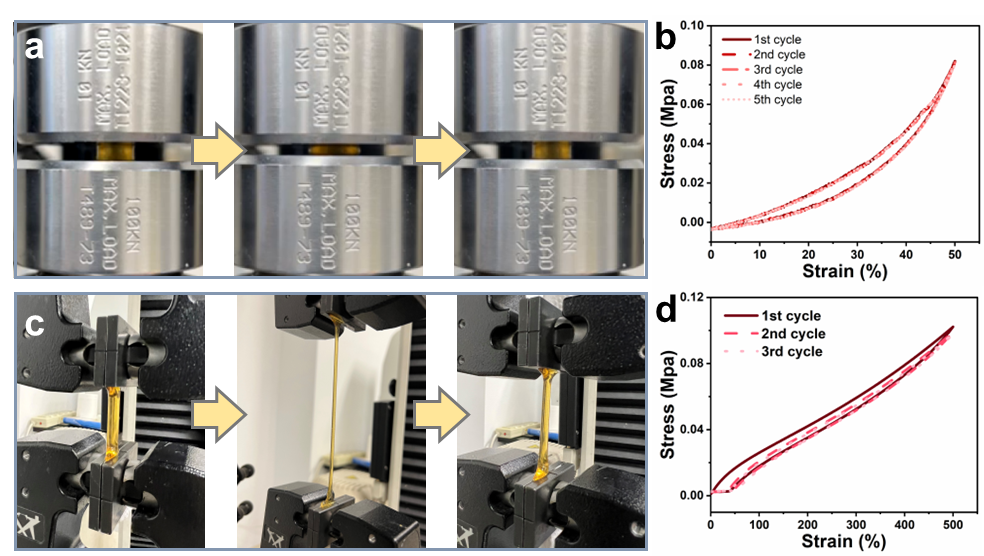


**Figure S10.** Cyclic compression and tensile mechanical properties of CPA. (a) Photographic depiction of the appearance during cyclic compression testing in loaded and unloaded states. (b) Load-unload curves. (c) Photographic depiction of the appearance during cyclic tensile testing in loaded and unloaded states. (d) Load-unload curves.


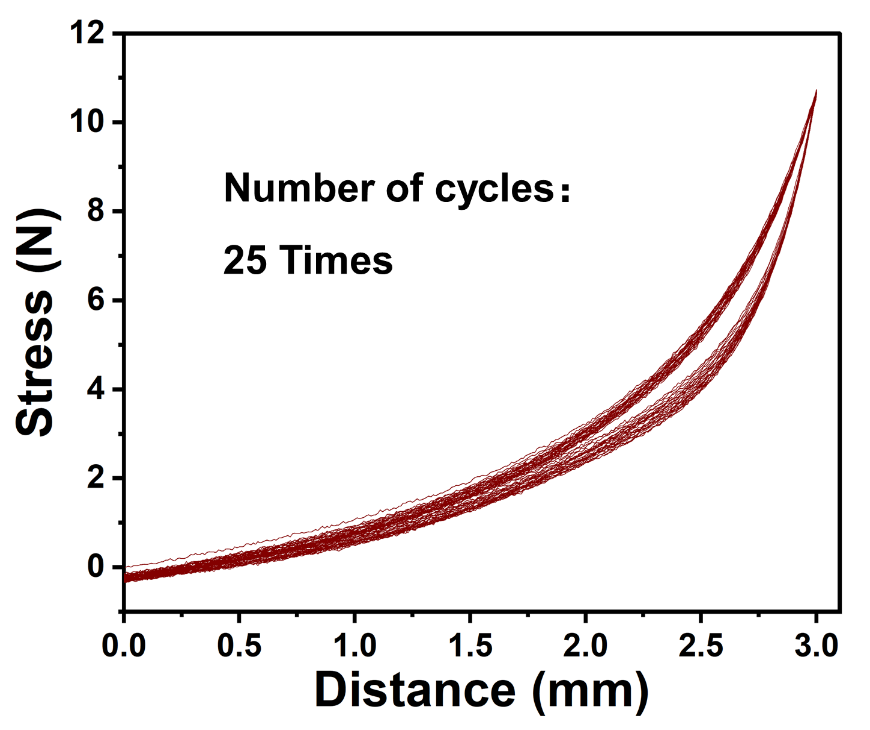


**Figure S11.** Cyclic compression testing of CPA Hydrogels (n=5, number of cycles: 25 times).


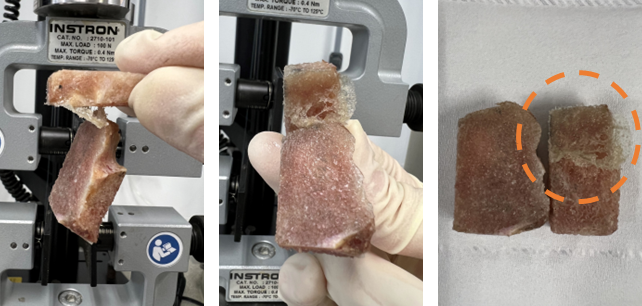


**Figure S12.** Appearance of CPA remaining on the bone surface after bond disruption (orange circle indicating the film formed from CPA).


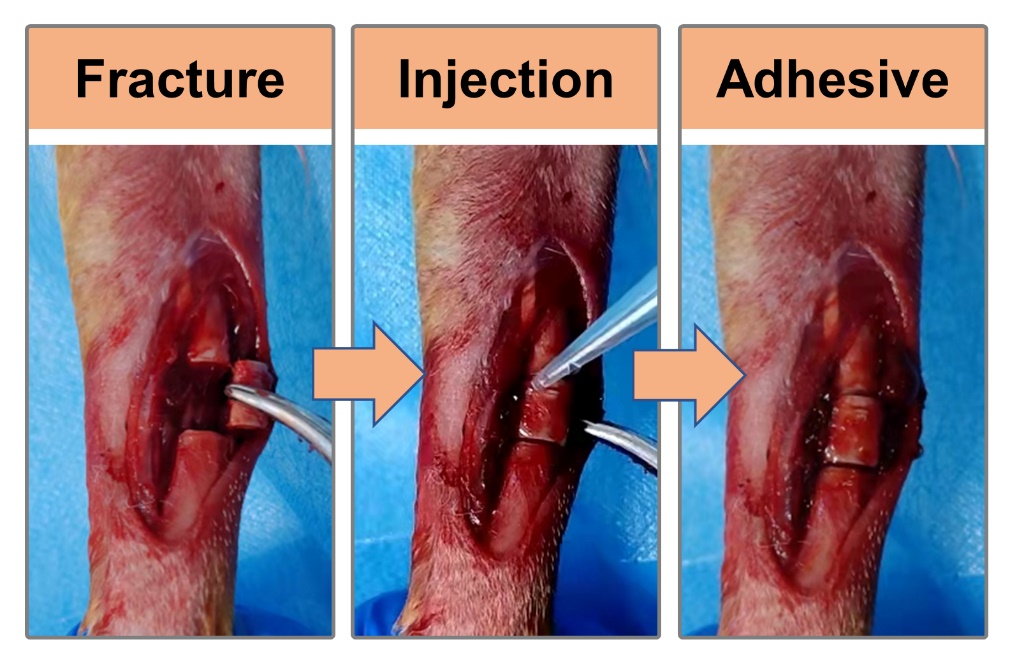


**Figure S13.** Photographs of comminuted radial fractures in rabbits and bone adhesion after CPA injection.


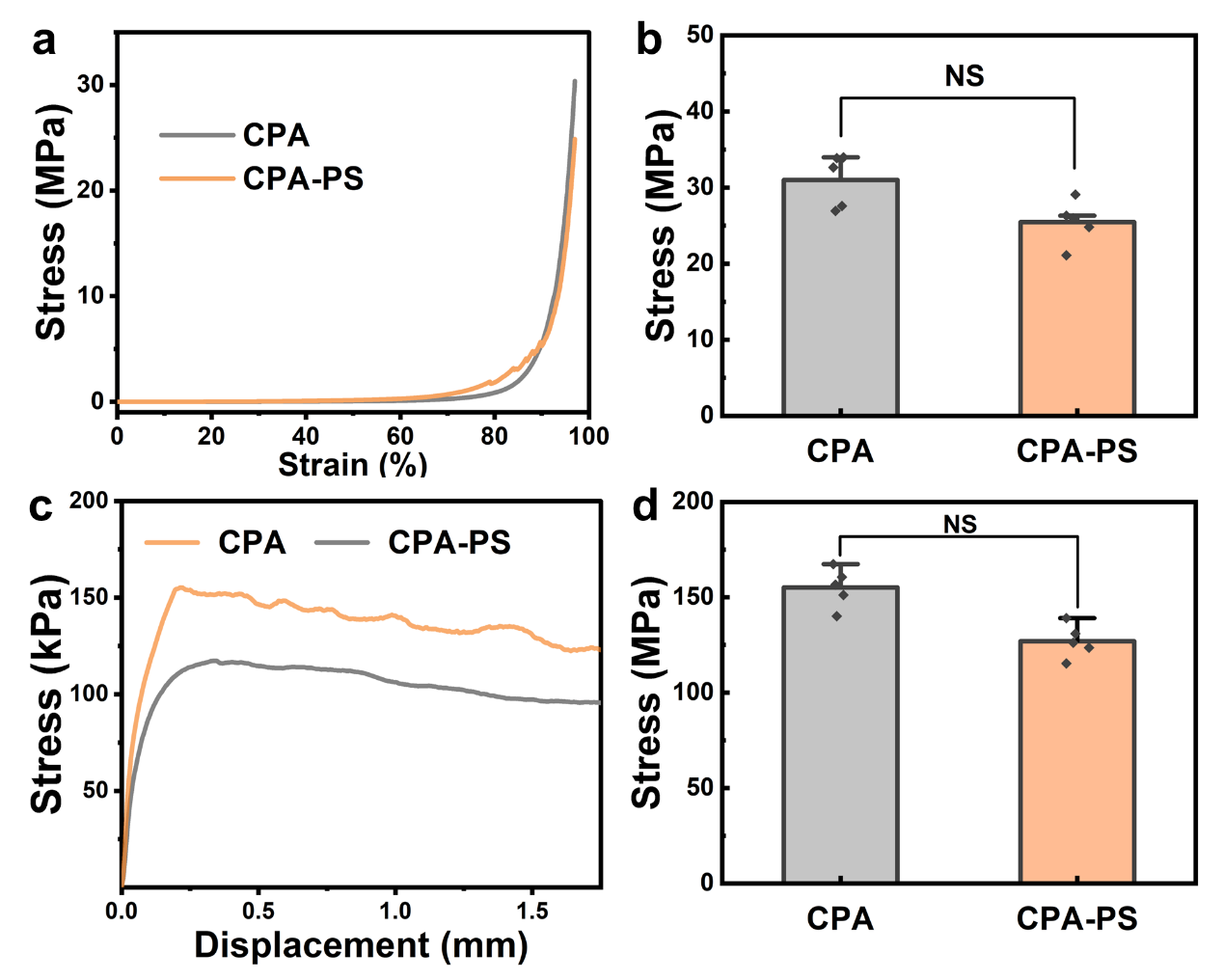


**Figure S14.** Post-swelling mechanical performance of CPA hydrogels. Compressive stress-strain curves (a) and stress quantification (b) before and after 24 h PBS immersion (n=5). Adhesive stress-strain curves (c) and stress quantification (d) comparison of CPA-bonded bone fragments pre- and post-immersion (n=5). (CPA-PS represents post-swelling CPA hydrogels, data are presented as mean ± SD, *p < 0.05, **p < 0.01, ***p < 0.001, NS: no significant difference)


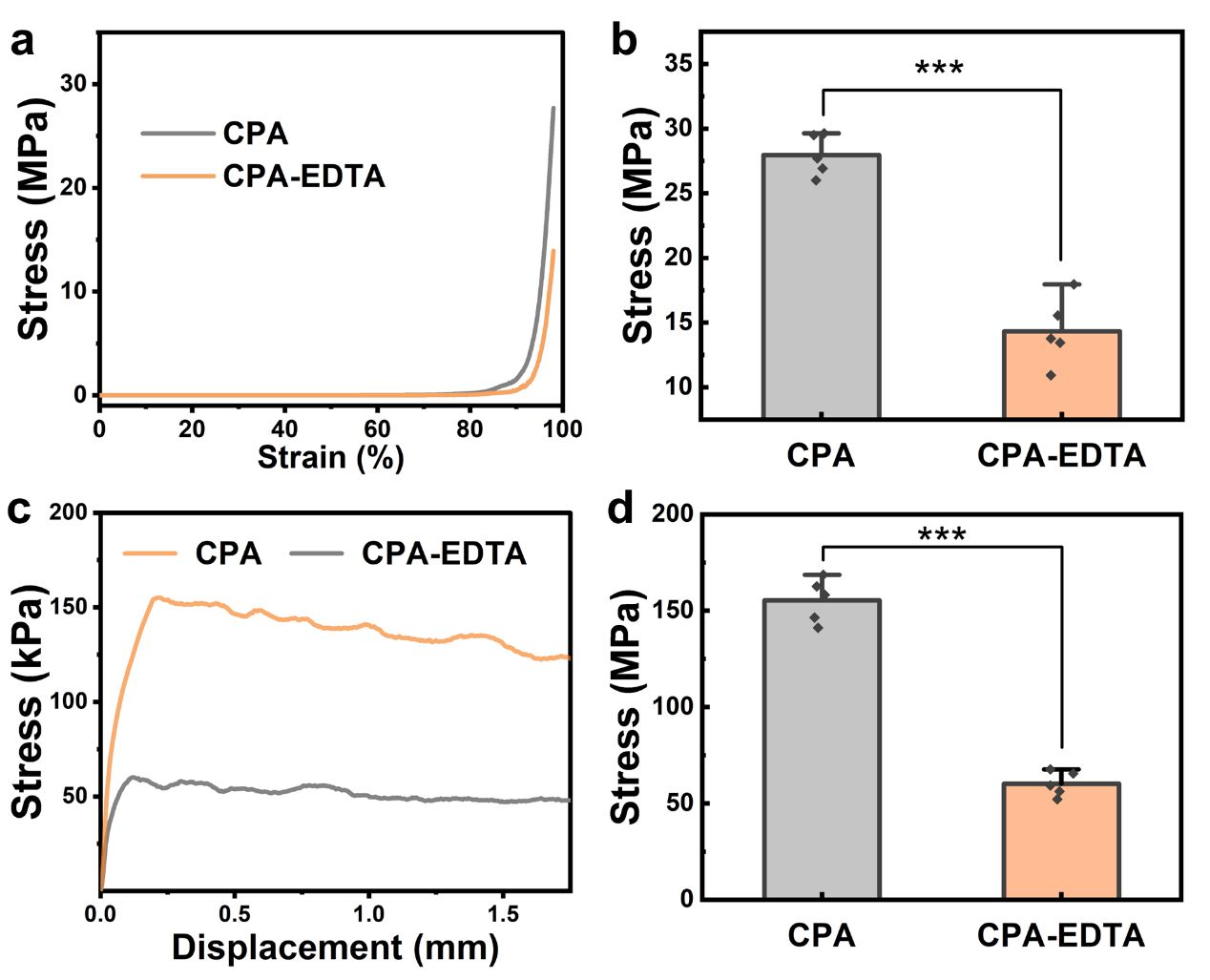


**Figure S15.** Mechanical performance of EDTA-treated CPA hydrogels. Compressive stress-strain curves (a) and stress quantification (b) with and without EDTA treatment (n=5). Adhesive stress-strain curves (c) and stress quantification (d) comparison of CPA-bonded bone fragments with and without EDTA treatment (n=5). (CPA-EDTA represents EDTA-treated CPA hydrogels, data are presented as mean ± SD, *p < 0.05, **p < 0.01, ***p < 0.001, NS: no significant difference)


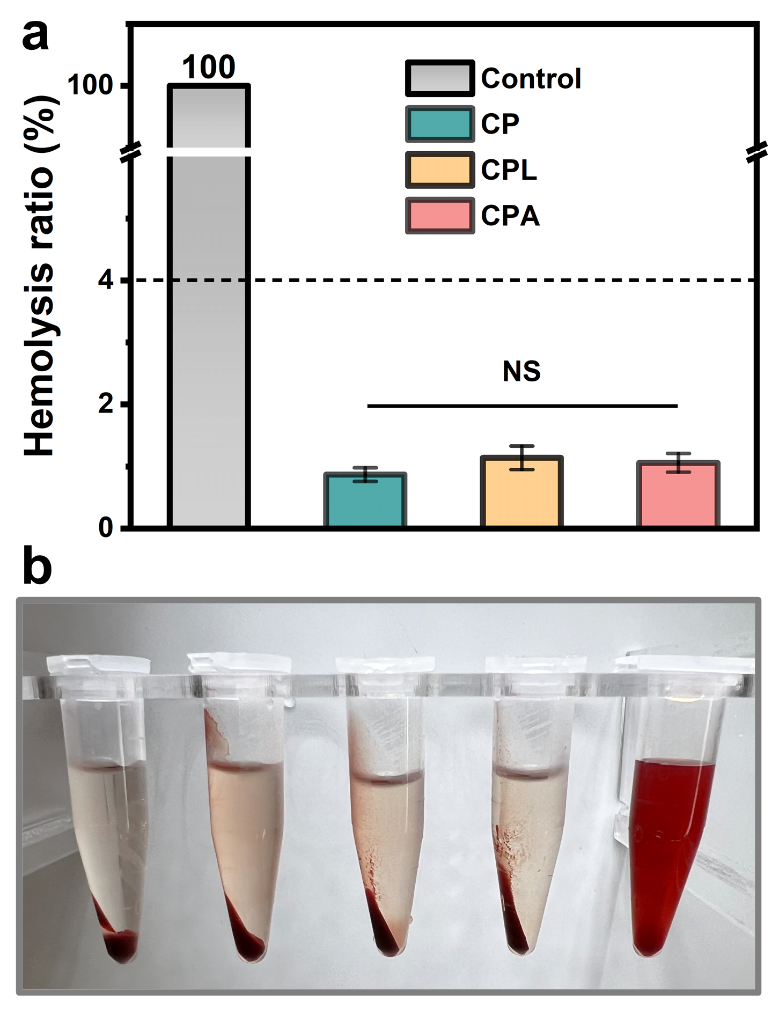


**Figure S16.** (a) Quantitative diagram of the hemolysis experiment. (b) Pictures of hemolysis experiment. From left to right: blank control group (0.9% saline), CP group, CPL group, CPA group, and positive control group (Triton X-100).


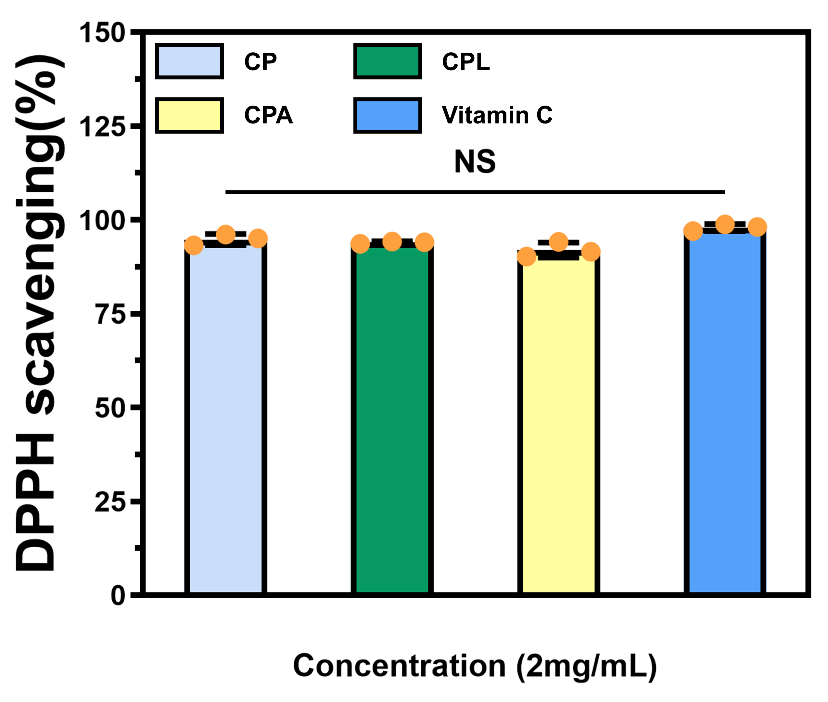


**Figure S17.** Quantification of DPPH clearance of the bone adhesives.


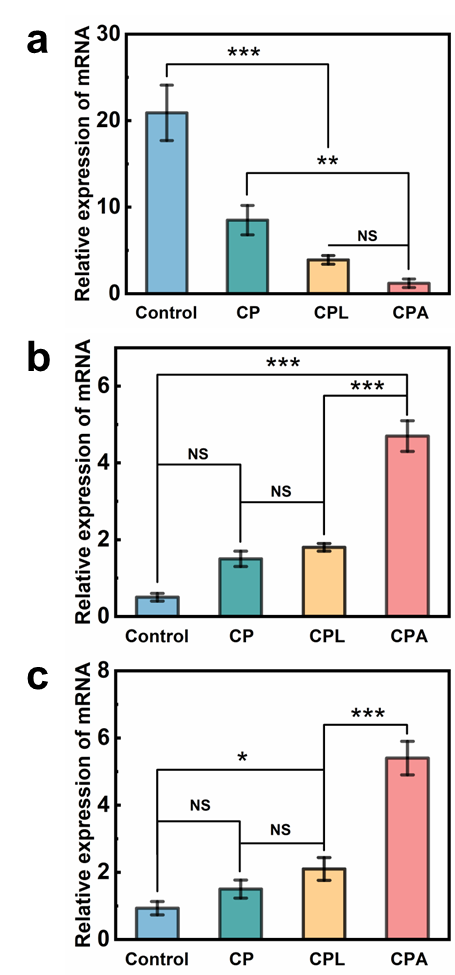


**Figure S18.** (a) CD68, (b) CD31, and (c) CD206 gene expression of macrophages.


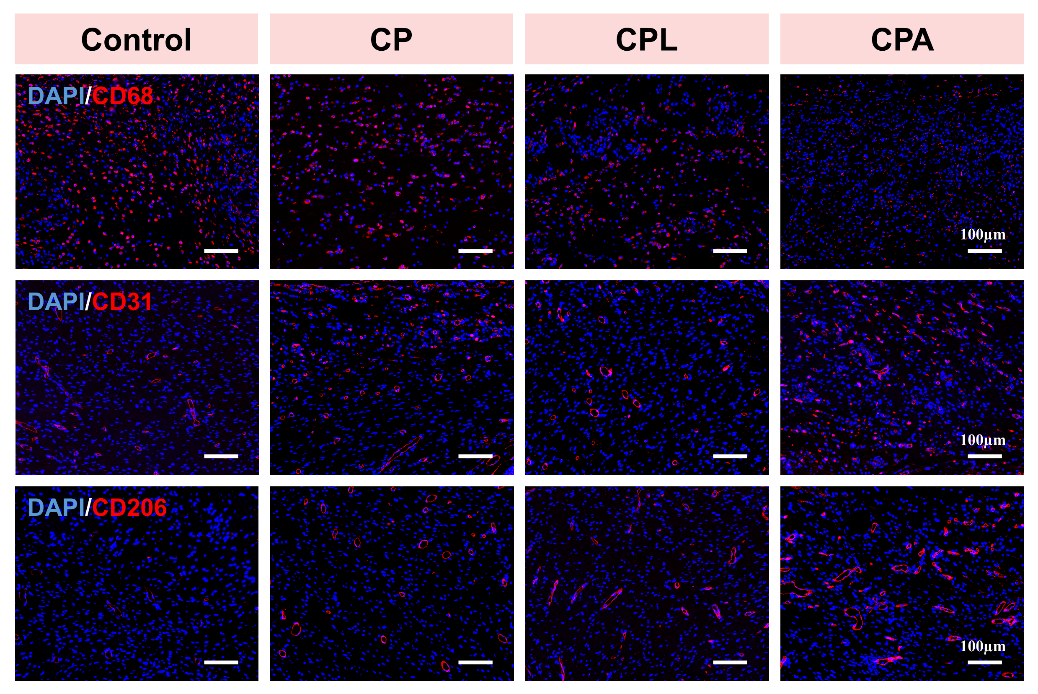


**Figure S19.** Fluorescence microscopy images of immunofluorescent staining of CD68, CD31 and CD206. Scale bar: 100 μm.


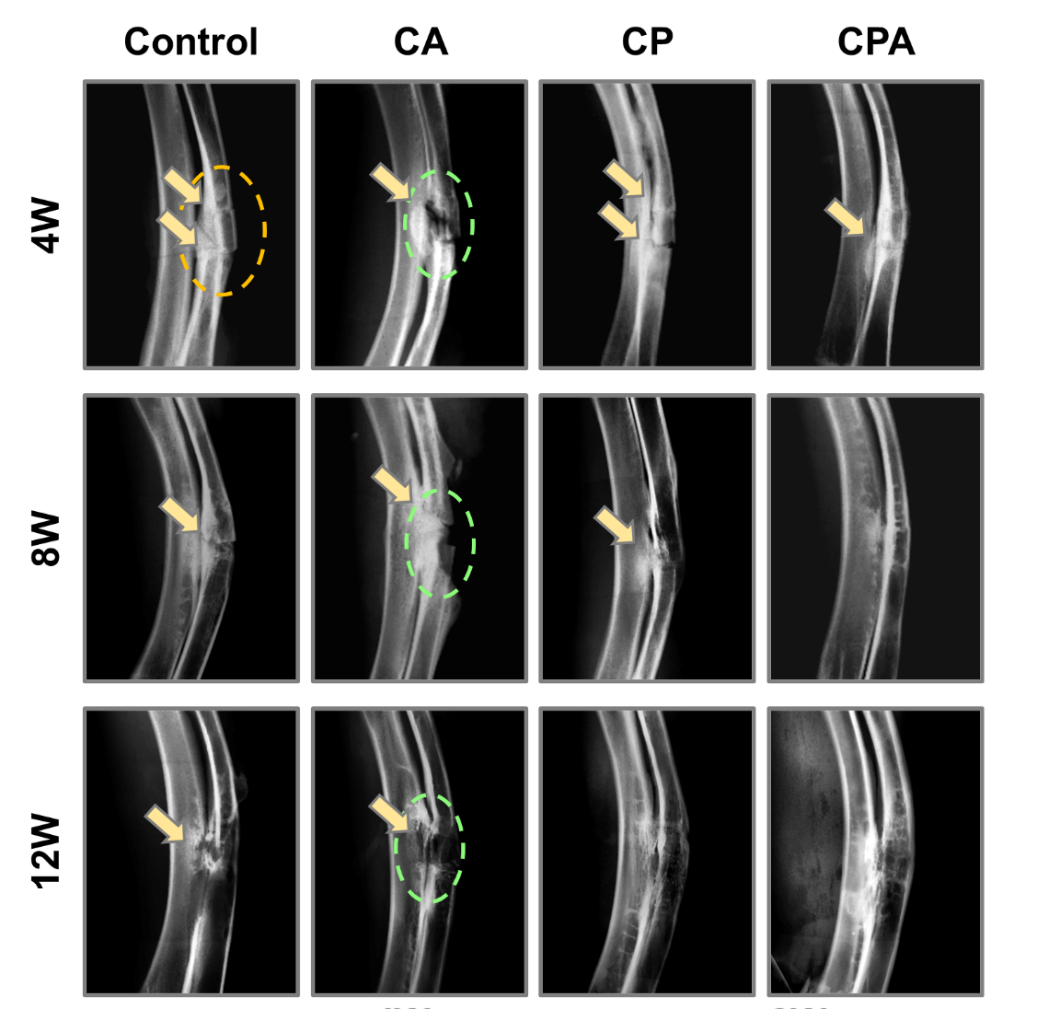


**Figure S20.** Representative X-ray images of the fracture site at 4, 8 and 12 weeks postoperatively (yellow circles indicating the fracture dislocation; yellow arrows indicating the fracture line; green circles marking the bone defect).
